# Senescence‐Driven IL‐17A Inflammatory Circuit Promotes Epithelial–Mesenchymal Transition (EMT) and Progression in Age‐Related Posterior Subcapsular Cataracts

**DOI:** 10.1111/acel.70456

**Published:** 2026-03-24

**Authors:** Yan Ni, Yu Ma, Zewen Ren, Yi Zhu, Liangping Liu, Runping Duan, Lu Qin, Yang Chen, Rong Ju, Yingyan Qin, Mingxing Wu

**Affiliations:** ^1^ State Key Laboratory of Ophthalmology, Zhongshan Ophthalmic Center, Sun Yat‐Sen University, Guangdong Provincial Key Laboratory of Ophthalmology and Visual Science Guangzhou China; ^2^ Guangdong Provincial Clinical Research Center for Ocular Diseases Guangzhou China; ^3^ Department of Laboratory Medicine and Pathology Mayo Clinic Rochester Minnesota USA

**Keywords:** cellular senescence, epithelial–mesenchymal transition, lens epithelial cells, posterior subcapsular cataracts, senescence‐associated secretory phenotype

## Abstract

Cataract is a leading cause of visual impairment worldwide, and its prevalence is increasing with population aging. The majority are age‐related cataracts (ARC), clinically classified into nuclear, cortical, and posterior subcapsular cataracts (PSC) by opacity location. Mechanisms of cataractogenesis remain incompletely understood. While cortical and nuclear cataracts are largely attributed to crystallin aggregation, such protein‐centric mechanisms fail to explain the early onset and axial location of PSC. Morphologically, PSC resembles posterior capsule opacification (PCO), a secondary cataract driven by epithelial–mesenchymal transition (EMT) of lens epithelial cells (LECs), suggesting LEC‐EMT may also contribute to PSC. Using clinically stratified human lens samples, we confirmed EMT marker expression across PSC subtypes. Transcriptomic profiling revealed that LECs, the lens's sole metabolically active cells, in age‐related PSC (ARC‐PSC), compared with age‐related nuclear cataract (ANC) and clear lenses, exhibit elevated EMT signatures tightly linked to senescence‐associated inflammatory signaling. Aqueous humor (AqH) profiling demonstrated a pro‐inflammatory milieu across PSC subtypes, with IL‐17A uniquely elevated in ARC‐PSC, consistent with transcriptomic findings. Integrated analyses support a model in which senescence functions as an upstream driver, whereby senescent LECs release SASP factors, including IL‐17A, that activate NF‐κB signaling to amplify inflammation, reinforce senescence, and drive EMT. In vitro, senolysis and IL‐17A blockade disrupted this loop, attenuating senescence‐ and EMT‐associated phenotypes. Collectively, our study demonstrates that senescent LECs sustain an IL‐17A–NF‐κB circuit that drives EMT and accelerates ARC‐PSC progression, positioning ARC‐PSC as a unique human context to study senescence‐induced epithelial remodeling in aging tissues.

## Graphical abstract

Using multi‐region human PSC samples, we identified EMT as a consistent pathological feature. Mechanistically, senescent LECs secrete IL‐17A, a representative SASP factor, which activates NF‐κB signaling. NF‐κB further amplifies SASP production, thereby establishing a senescence–inflammation feedback loop that drives EMT, sustains pathological remodeling, and accelerates PSC progression.

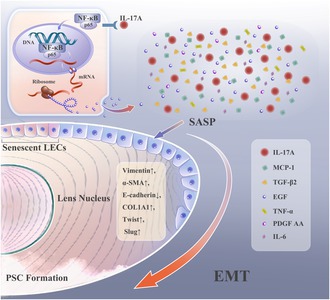

## 1 Introduction

Cataracts are a leading cause of blindness globally, affecting over 79 million individuals above age 50, a burden expected to rise with global aging (Cicinelli et al. 2023; Ehrlich et al. 2022). Among all cataracts, the three major clinical subtypes are nuclear, cortical, and posterior subcapsular cataracts (PSC), with age‐related cataracts (ARC) accounting for the majority of cases. Clinically, PSC may occur in combination with cortical and nuclear opacities or present as an isolated subtype, but in either case it causes disproportionately early and pronounced visual impairment due to its axial location. In addition, PSC poses greater surgical challenges, including higher risk of posterior capsule rupture and postoperative Nd:YAG capsulotomy (Levy‐Clarke et al. 2024). Despite its clinical significance, the mechanistic basis of PSC remains underexplored compared to other subtypes.

Mechanistically, most cataract research to date has focused on nuclear and cortical subtypes, where post‐translational modifications (PTMs) of lens crystallins serve as a primary explanatory model (Harding 2002; Schey et al. 2020). Under oxidative stress, mitochondrial dysfunction, aging, and epigenetic changes, crystallins undergo various PTMs including oxidation (R. J. W. Truscott and Friedrich 2019), glycation (Kyselova et al. 2004), deamidation (Lyon et al. 2019), phosphorylation(K. Zhang et al. 2018), truncation (Lin et al. 2013), racemization (Fujii et al. 2011), acetylation (DiMauro et al. 2014), and methylation (R. J. Truscott et al. 2012), leading to protein unfolding, reduced solubility, and aggregation. These changes ultimately disrupt lens architecture and optical clarity, resulting in lens opacification (Schey et al. 2020). However, this protein‐centric model does not fully explain the distinctive anatomical and clinical features of PSC. Typically presenting as shallow, plaque‐like opacities in the posterior subcapsular cortex—often described as grainy or “crust‐like”—PSCs may occur independently or coexist with nuclear and cortical cataracts. Their unique morphology and rapid progression suggest that additional, non‐protein‐based pathological processes may be involved.

Notably, based on our long‐term clinical observations, PSC exhibit striking morphological similarities to posterior capsule opacification (PCO)—a secondary cataract widely attributed to epithelial–mesenchymal transition (EMT) (Qin et al. 2017). EMT, a process in which epithelial cells lose polarity and acquire mesenchymal‐like properties, has been proposed as a pathological feature of PSC, based on the observation of backward migrating LECs in transmission electron microscopy studies (Eshaghian and Streeten 1980). However, the initial molecular signals that trigger EMT in PSC remain poorly defined.

Lens epithelial cells (LECs), the only metabolically active cell population in the lens, maintain lens transparency and structural integrity by regulating redox balance, ion transport, and crystallin biosynthesis (Z. Liu et al. 2023; Nandi et al. 2019). Anatomically located beneath the anterior capsule, LECs are particularly vulnerable to disturbances in their surrounding microenvironment, which encompasses both intrinsic and extrinsic factors. Notably, the intrinsic environment—comprising aging, oxidative stress, and metabolic imbalance—not only drives PTMs of crystallins, but also induces DNA damage and promotes cellular senescence (Z. Wu et al. 2024), a process increasingly recognized in cataractogenesis and lens aging pathology (Gulluni et al. 2021). Meanwhile, the extrinsic environment, primarily represented by the aqueous humor (AqH), constitutes the metabolic interface of LECs and contains inflammatory and immune‐modulatory cytokines, some of which overlap with known senescence‐associated secretory phenotype (SASP) factors (X. Li et al. 2023). SASP components have been shown to promote EMT in neighboring cells, further amplifying local tissue remodeling (Chambers et al. 2021). However, these findings have largely been confined to cancer and fibrotic models, and whether a senescence‐driven microenvironment actively promotes EMT in the human lens remains unexplored. Such a possibility is particularly intriguing given the lens's closed anatomy, which may allow paracrine SASP signals to accumulate and induce sustained epithelial remodeling.

Using a large cohort of clinically stratified human PSC samples, we confirmed EMT as a consistent pathological feature across subtypes. To explore its upstream regulation, we focused on ARC‐PSC—a subtype with minimal inflammatory interference—for transcriptomic and mechanistic analyses. Our results demonstrate that cellular senescence plays a central role in driving EMT through a paracrine signaling loop. These findings reveal a previously unrecognized mechanism in human samples by which senescence‐associated changes in LECs initiate pathological remodeling. Given the well‐defined epithelial structure of the lens and the clinical accessibility of ARC‐PSC specimens, our study highlights ARC‐PSC not only as a lens pathology but also as a tractable human platform for studying senescence‐induced epithelial remodeling in aging tissues.

## 2 Materials and Methods

### 2.1 Study Design and Subject Recruitment

Patients were consecutively enrolled from the Zhongshan Ophthalmic Center, Sun Yat‐sen University (Guangzhou, China), between September 2023 and September 2024. All enrolled patients underwent standard preoperative ocular examinations and systemic blood tests. Eligible participants included adults diagnosed with cataracts due to age‐related and non‐age‐related causes, including high myopia (HM), prior pars plana vitrectomy (PPV), long‐term glucocorticoid use, or diabetes mellitus. Exclusion criteria included prior ocular surgery (except PPV), coexisting ocular diseases (e.g., glaucoma, uveitis), and systemic disorders such as hypertension.

Cataract severity and subtype were graded according to a modified version of the Lens Opacities Classification System III (LOCS III) (Chylack et al. 1993), with posterior subcapsular opacity scored from P1 to P5. PSC diagnosis was established based on posterior opacity predominance on LOCS III grading and intraoperative assessment. Two independent ophthalmologists blinded to clinical diagnoses performed grading to ensure objectivity.

The control group comprised patients with age‐related nuclear cataracts (ANC), no systemic disease, and scheduled for routine phacoemulsification with intraocular lens implantation.

### 2.2 Sample Collection

All cataract surgeries were performed by a single surgeon (M.W.) to ensure procedural consistency. AqH samples (100–200 μL) were obtained at the onset of surgery via anterior chamber paracentesis using a 30‐gauge needle and stored at −80°C.

Anterior lens capsule specimens were obtained through continuous curvilinear capsulorhexis during phacoemulsification. Posterior subcapsular cortex samples were collected following ultrasonic emulsification and aspiration of the lens nucleus using a manually operated cannula (patented by Mingxing Wu et al. ZL 2022 22,351,418.X). The collected lens material was centrifuged, and the precipitates were retained and stored at −80°C.

Transparent human lenses were obtained from the Guangdong Eye Bank as normal controls. Donors had no documented history of cataract or systemic illness, as outlined in Table S1. For comparability with cataract specimens, only the central anterior capsule and posterior subcapsular cortex were dissected.

### 2.3 RNA Sequencing and Bioinformatics Analysis

Total RNA was extracted with TRIzol, quantified by NanoDrop, and quality‐checked on an Agilent Fragment Analyzer. Samples with RIN ≥ 7.0 and yield > 400 ng were used for library preparation (three biological replicates per group). Libraries were generated using NEBNext Ultra RNA Library Prep Kit and sequenced on an Illumina NovaSeq 6000. Clean reads were aligned to the human reference genome with HISAT2 (v2.0.5), and gene counts were obtained using FeatureCounts (v1.5.0‐p3). Differential expression analysis was performed with DESeq2 (v1.20.1), and GO enrichment of significant DEGs (*p* < 0.05) was conducted using clusterProfiler. Data visualization was generated with Bioinformatics and OmicStudio online platforms.

Detailed information on reagents, kits, and instruments is provided in Table S2.

### 2.4 Western Blot Assay

Proteins were extracted with lysis buffer, quantified by BCA assay, separated by SDS–PAGE, and transferred to PVDF membranes. Membranes were incubated with primary and HRP‐conjugated secondary antibodies, and signals were visualized by ECL. Band intensities were quantified using ImageJ (v1.53t). Antibody information is provided in Table S3.

### 2.5 Quantitative Real‐Time PCR (qRT‐PCR)

Total RNA was extracted using the RNAiso reagent and reverse transcribed into cDNA using the PrimeScript RT Master Mix Kit according to the manufacturer's instructions. qRT‐PCR was conducted using the Taq Pro Universal SYBR Green qPCR Master Mix on a LightCycler 480 system. Primer sequences are listed in Table S4.

### 2.6 Inflammatory Cytokine Quantification

Inflammatory cytokines (TGF‐β2, IL‐6, TNF‐α, EGF, PDGF‐AA) were quantified using MILLIPLEX MAP magnetic bead panels on a Luminex platform (25 μL/well). MCP‐1 and IL‐17A concentrations were measured with specific enzyme‐linked immunosorbent assay (ELISA) kits following manufacturer's protocols.

### 2.7 Immunofluorescence (IF) Staining

Samples were fixed, permeabilized, blocked, and incubated with primary and fluorescent secondary antibodies. Nuclei were counterstained with DAPI. Images were acquired using a Zeiss LSM880 laser scanning confocal microscope and processed with Zen software (Zeiss). IF signal intensity was quantified using ImageJ software (v1.53t). Antibody details are provided in Table S3.

### 2.8 Scanning Electron Microscopy (SEM)

Anterior lens capsules were fixed in glutaraldehyde, post‐fixed in osmium tetroxide, dehydrated, and gold‐coated. Imaging was performed using a Hitachi SU8010 scanning electron microscope at 5 kV acceleration voltage. Surface morphology of LECs was analyzed at 1000× to 5000× magnifications.

### 2.9 Correlation Analysis

Spearman's rank correlation was applied to assess associations between gene expression (senescence and EMT markers) and cataract severity. Subgroup analyses within PSC subtypes were included when relevant. Analyses were performed in GraphPad Prism (v9.0.0), with correlation coefficients (*r*) and significance values (*p*) reported.

### 2.10 Cell and Ex Vivo Lens Capsule Culture and Treatments

The human LEC line SRA01/04 (RRID: CVCL_7157) was kindly provided by Prof. Fu Shang (State Key Laboratory of Ophthalmology, Guangzhou, China) and cultured in Dulbecco's Modified Eagle Medium (DMEM) with 10% FBS at 37°C, 5% CO_2_. Mycoplasma contamination was routinely tested by a LAMP‐based assay and consistently found negative.

For stimulation, cells were exposed to recombinant IL‐17A (200 ng/mL, 24 h) or oxidative stress induced by H_2_O_2_ (200 μM, 2 h) or glucose oxidase (30 mU/mL, 2 h), followed by 24 h in fresh DMEM. Conditioned media were collected and applied to untreated LECs for 24 h to assess paracrine effects.

For intervention, cells were treated with PCC1 (20 μM) or Secukinumab (70 nM) for 24 h.

For ex vivo culture, freshly isolated human anterior lens capsules were flattened with the epithelial side facing upward and incubated in DMEM with 20% FBS at 37°C for 48 h. IL‐17A, PCC1, or Secukinumab were applied at the same concentrations as in vitro.

### 2.11 Cell Counting Kit‐8 (CCK‐8)

Cell viability was assessed using the CCK‐8 according to the manufacturer's instructions. Absorbance at 450 nm was measured using a Synergy H1 Hybrid Reader.

### 2.12 Wound Healing Assay

Confluent cell cultures were scratched with a pipette tip and incubated in serum‐free medium with or without treatment. Scratch images were captured at 0, 6, 12, and 24 h using a Leica Dmi1 inverted microscope. The width of the scratch area was measured and quantified using ImageJ software (v1.53t).

### 2.13 SA‐β‐Galactosidase (SA‐β‐gal) Staining

SA‐β‐gal activity was detected using a commercial kit following the manufacturer's instructions. At least five random fields per well were analyzed, and the percentage of SA‐β‐gal‐positive (blue) cells relative to total cells was calculated.

### 2.14 Statistical Analysis

All experiments were performed with ≥ 3 independent biological replicates. Statistical analyses were conducted using GraphPad Prism (v9.0.0).

Two‐group comparisons were analyzed by unpaired two‐tailed Student's *t*‐test, while multiple group comparisons were assessed by one‐way ANOVA with appropriate post hoc tests. Data normality was evaluated with the Shapiro–Wilk test; normally distributed datasets were analyzed using Fisher's least significant difference (LSD) test, and non‐normally distributed datasets with Tamhane's T2 test. Results are shown as mean ± SEM, with statistical significance defined as *p* < 0.05.

## 3 Results

### 3.1 EMT Is a Consistent Pathological Feature in the Lens Specimens of the PSC Patients

**FIGURE 1:**
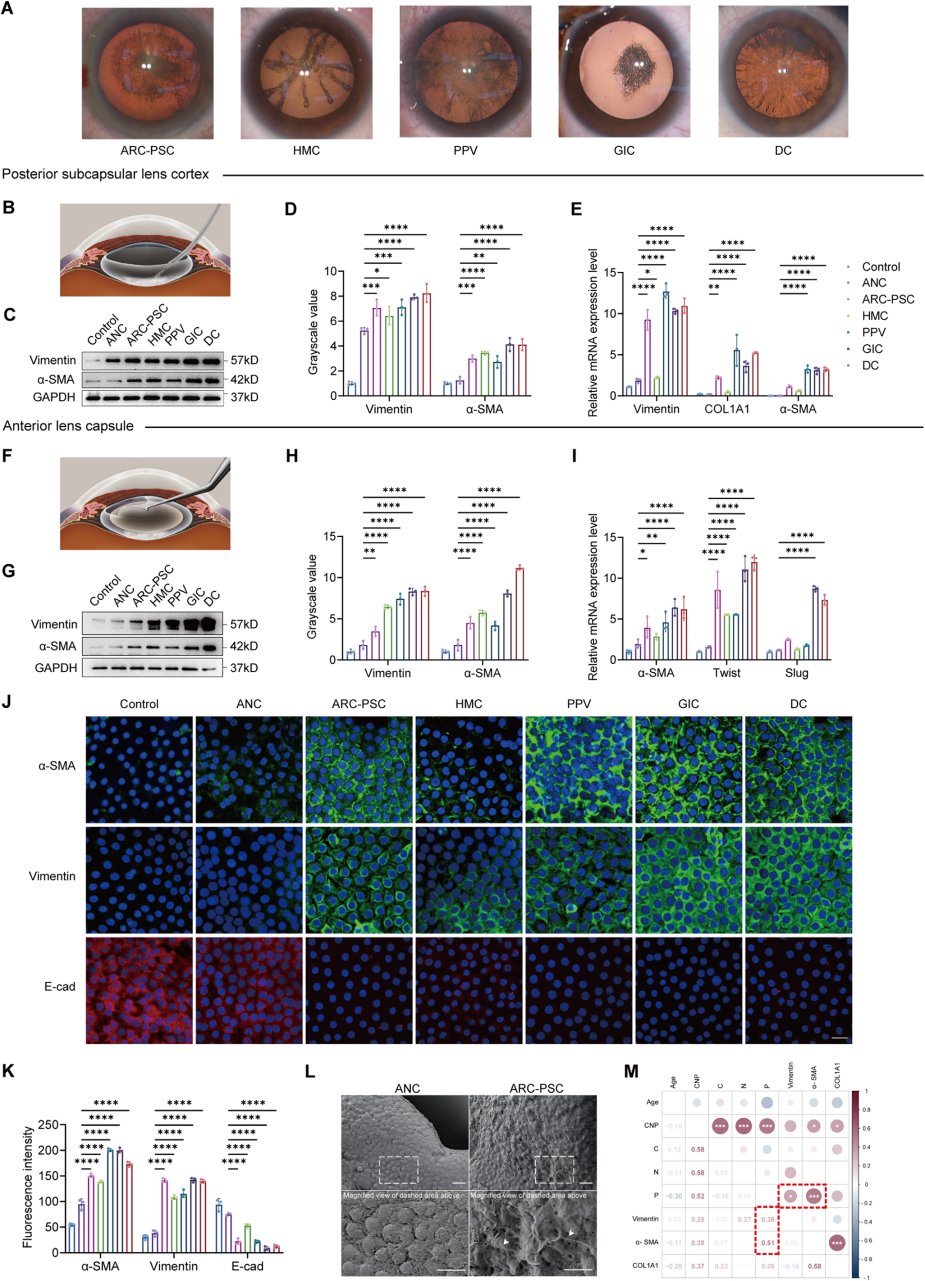
EMT activation in the posterior subcapsular cortex and anterior lens epithelium in posterior subcapsular cataracts (PSC) patients. (A) Representative intraoperative retroillumination photographs illustrating PSCs with different etiologies. (B) Schematic diagram of intraoperative sampling of posterior subcapsular cortex. (C, D) Western blot (C) and densitometric analysis (D) of Vimentin and α‐SMA protein levels in the posterior subcapsular cortex. (E) Quantitative real‐time PCR (qRT‐PCR) analysis of Vimentin, COL1A1, and α‐SMA mRNA expression in posterior subcapsular cortex samples. (F) Schematic diagram of intraoperative sampling of anterior lens capsules. (G, H) Western blot (G) and densitometric analysis (H) of Vimentin and α‐SMA protein levels in anterior lens capsules. (I) qRT‐PCR analysis of α‐SMA, Twist1, and Slug mRNA expression in anterior lens capsules. (J, K) Representative immunofluorescence (IF) images (J) and quantification (K) of anterior lens capsules stained for Vimentin (green), α‐SMA (green), and E‐cadherin (red). DAPI (blue) was used for nuclear counterstaining. Scale bar: 20 μm. (L) Scanning electron microscopy images of anterior lens epithelial surfaces from age‐related nuclear cataract (ANC) and age‐related cataracts (ARC)‐PSC samples. Dashed boxes indicate regions shown at higher magnification below. Compared with ANC, ARC‐PSC exhibits increased surface irregularity and filamentous protrusions (arrowheads). Scale bar: 20 μm. (M) Spearman correlation analysis between posterior subcapsular opacity grade and mRNA expression levels of epithelial–mesenchymal transition (EMT)‐related genes. Data are presented as mean ± SEM. *n* = 3 biologically independent experiments. One‐way ANOVA was used for multiple comparisons. *p < 0.05, **p < 0.01, ***p < 0.001, ****p < 0.0001.

While previous studies have hinted at epithelial changes in PSC, it remains unclear whether EMT represents a consistent pathological hallmark across its subtypes. To address this, we harvested 292 clinical lens specimens across five PSC subtypes: age‐related PSC (ARC‐PSC), highly myopic cataract (HMC), post‐pars plana vitrectomy (PPV) cataract, glucocorticoid‐induced cataract (GIC), and diabetic cataract (DC). Age‐related nuclear cataracts (ANC) were chosen as a disease control, while transparent donor lenses served as normal references. Demographic details of each group are summarized in Table 1. LOCS III grading confirmed elevated posterior subcapsular opacities in all PSC subtypes (Figure 1A).

**TABLE 1:** Clinical features of the study subjects.

Characteristics	ANC	ARC‐PSC	HMC	PPV	GIC	DC
Numbers	64	66	47	39	26	50
Age (years, *p*)	70.2 ± 10.3	67.1 ± 8.2, 0.125	56.4 ± 11.0, < 0.0001	57.0 ± 9.7, < 0.0001	46.5 ± 18.6, < 0.0001	67.8 ± 12.9, 0.281
Male/Female	41/23	31/35	27/20	24/15	15/11	20/30
LOCS III (posterior subcapsular, *p*)	1.7 ± 0.8	3.4 ± 0.5, < 0.0001	2.1 ± 0.8, 0.014	3.0 ± 0.8, < 0.0001	3.2 ± 0.8, < 0.0001	3.3 ± 0.7, < 0.0001

*Note:* LOCS III, the Lens Opacities Classification System III. Data are means ± SEM. One‐way ANOVA and Fisher's least significant difference procedure were used to identify significant differences compared to the ANC group.

We first examined the pathological focus of PSC—the opaque posterior subcapsular cortex (Figure 1B). Western blot revealed a significant increase of Vimentin and α‐smooth muscle actin (α‐SMA) in the opaque posterior subcapsular cortex of all PSC groups—both age‐related and complicated—compared to the control and ANC groups (Figure 1C,D). Consistently, quantitative real‐time PCR (qRT‐PCR) confirmed higher expression of Vimentin, collagen type I alpha 1 chain (COL1A1), and α‐SMA in PSC samples (Figure 1E), indicating consistent EMT activation within the lesion site of PSC.

To determine whether these changes originated from the lens epithelium, we next analyzed anterior lens capsules, where viable LECs reside (Figure 1F). Western blot analyses of anterior lens capsules showed elevated Vimentin and α‐SMA across all PSC subtypes (Figure 1G,H), while qRT‐PCR further showed increased α‐SMA and Twist1 expression. The GIC and DC groups additionally exhibited elevated Slug levels (Figure 1I), suggesting subtype‐dependent variation in EMT activation.

Because bulk assays cannot precisely resolve the fraction of LECs undergoing EMT, we further performed immunofluorescence (IF) staining to spatially resolve marker expression. IF confirmed EMT activation in all PSC subtypes, characterized by increased α‐SMA and Vimentin expression alongside reduced E‐cadherin levels (Figure 1J,K). Clear EMT signals were evident in ARC‐PSC compared to age‐matched ANC controls, while GIC and DC samples exhibited the strongest activation.

Morphological assessment by scanning electron microscopy (SEM) revealed pronounced surface irregularity and filamentous protrusions distributed across the epithelial surface in ARC‐PSC anterior capsules, in contrast to the smooth and uniformly organized epithelial surface observed in ANC samples (Figure 1L).

Finally, Spearman correlation analyses showed that mRNA levels of Vimentin and α‐SMA positively correlated with posterior subcapsular opacity, while α‐SMA and COL1A1 also correlated with total lens opacification (Figure 1M), supporting a broader role for EMT in cataract progression beyond PSC.

Together, these molecular, spatial, and morphological findings position EMT as a consistent pathological feature of PSC. Given the minimal inflammatory confounders and strong senescence signature of ARC‐PSC, we selected this subtype as a tractable context for investigating EMT‐inducing mechanisms.

### 3.2 Transcriptomic Profiling Reveals Senescence‐Associated Signaling as an Upstream Regulator of EMT in PSC

**FIGURE 2:**
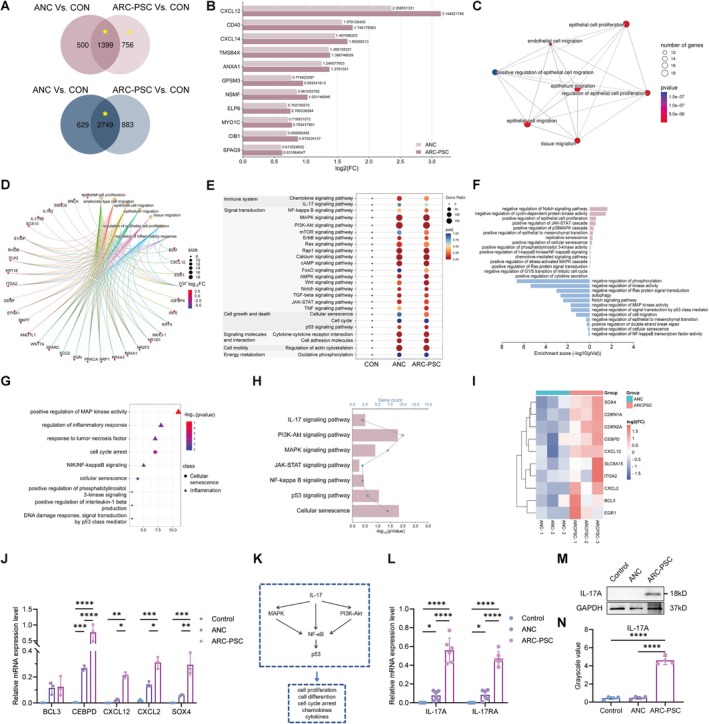
Transcriptomic profiling reveals epithelial–mesenchymal transition (EMT), inflammation, and senescence as key signatures in ARC‐PSC lens epithelial cells. (A) Venn diagram showing the number of upregulated (red) and downregulated (blue) differentially expressed genes (DEGs) in age‐related cataracts (ARC)‐posterior subcapsular cataract (PSC) versus control and age‐related nuclear cataract (ANC) versus control comparisons. (B) Bar graph of EMT‐related genes with fold change (FC) in log_2_ scale in the marked area (yellow triangle) in panel A. (C, D) gene ontology (GO) enrichment of shared upregulated DEGs between ANC and ARC‐PSC: Visualized using enrichment map (C) and gene‐concept network plot (D). (E) Dot plot for Kyoto Encyclopedia of Genes and Genomes (KEGG) enrichment results of DEGs across all three groups (control, ANC, ARC‐PSC). (F) Enrichment analysis of RNA‐seq data sets in the marked area (yellow star) in panel A. Bar graph of enriched terms of DEGs in the ARC‐PSC group, colored by *p*‐values and showing GO biological processes enrichment analysis of upregulated genes (right panel) and for downregulated genes (left panel). (G) Bubble plot representing enriched GO terms in the ARC‐PSC group. (H) Bar graph for KEGG enrichment results of upregulated DEGs shared between ANC and ARC‐PSC groups. (I) Heatmap for expression levels of SASP‐related DEGs between ANC and ARC‐PSC groups. (J) Quantitative real‐time PCR (qRT‐PCR) showing mRNA expression of SASP‐related DEGs in the anterior lens capsules. (K) Presumed signaling pathway: IL‐17 activates the MAPK and PI3K‐Akt pathways, indirectly or directly activating NF‐κB, which subsequently promotes the activation of p53. (L) qRT‐PCR showing mRNA expression of IL‐17A and IL‐17RA in the anterior lens capsules (*n* = 6 each). (M, N) Representative western blot images (M) and densitometric analysis (N) illustrating the protein expression levels of IL‐17A in anterior lens capsules (*n* = 4 each). All RNA‐seq analyses were conducted with 3 biological replicates per group. Validation experiments used independent clinical samples as indicated. Data are presented as mean ± SEM. One‐way ANOVA was used for multiple group comparisons. *p < 0.05, **p < 0.01, ***p < 0.001, ****p < 0.0001.

Given the consistent EMT activation observed in PSC, we next asked whether broader transcriptional programs could reveal upstream drivers. To address this, we performed RNA sequencing on anterior lens capsules from ARC‐PSC, ANC, and clear lenses (*n* = 3 per group; Figure 2A, Figure S1A). ARCs were selected to minimize confounding influences present in complicated cataracts.

Consistent with the EMT phenotypes observed in our tissue analyses, RNA sequencing revealed significant upregulation of EMT‐related genes specifically in ARC‐PSC samples (yellow triangle in Figure 2A), as visualized in Figure 2B. GO enrichment of ARC‐PSC‐specific differentially expressed genes (DEGs) highlighted a coherent network of biological processes including epithelial cell migration, epithelium and tissue migration, and regulation of epithelial cell proliferation, supported by enrichment of terms related to cell‐substrate adhesion and cytoskeletal organization (Figure 2C). Representative DEGs contributing to these processes included ITGA2, an integrin essential for epithelial adhesion and motility; KRT16, a stress‐responsive keratin associated with epithelial plasticity; and extracellular‐matrix‐related genes such as LAMC2 and COL16A1, indicating early EMT‐like epithelial remodeling. Complementary network analysis further revealed genes bridging epithelial remodeling with inflammatory or chemotactic signaling pathways (Figure 2D). These included CXCL12, a chemokine that promotes directed epithelial migration; transcriptional regulators such as IRF6 and PLK2, linked to epithelial proliferation and stress responses; and immune‐associated genes such as B2M and IL17RB, suggesting that inflammatory cues may be transcriptionally integrated into the epithelial remodeling program in ARC‐PSC.

Building on these EMT‐specific findings, we next explored the broader transcriptomic landscape to distinguish PSC‐specific alterations from age‐related background shifts. ANC and ARC‐PSC shared a subset of transcriptional changes (yellow stars in Figure 2A), and pathway enrichment of these overlapping DEGs indicated activation of oxidative stress, senescence, and inflammatory processes—signatures common to age‐related cataracts (Figure 2E,F).

To further resolve PSC‐specific programs, we directly compared ARC‐PSC with ANC samples. GO analysis highlighted unique upregulation of senescence‐associated and inflammatory processes in ARC‐PSC (Figure 2G). Pathway analysis further identified enrichment of IL‐17, NF‐κB, PI3K‐Akt, and MAPK signaling pathways (Figure 2H).

The co‐occurrence of senescence‐ and inflammation‐ associated signatures in ARC‐PSC prompted us to investigate whether senescent LECs might actively contribute to the sustained inflammatory milieu. Heatmap analysis revealed substantial upregulation of canonical senescence‐associated secretory phenotype (SASP) factors—including CEBPD, CXCL2, CXCL12, and SOX4—in ARC‐PSC compared to ANC (Figure 2I), findings further validated by qRT‐PCR in clinical samples (Figure 2J). These findings support the presence of pro‐inflammatory senescent LECs in the PSC lens epithelium, establishing a microenvironment conducive to EMT activation.

Given the observed SASP activation and enrichment of pro‐inflammatory pathways, we next sought to identify specific mediators that could functionally link senescence to EMT. Among these, IL‐17A emerged as a compelling candidate. While IL‐17 has not previously been implicated in cataract pathogenesis, it is a well‐established mediator of chronic inflammation (Huangfu et al. 2023; Solá et al. 2023; Wang et al. 2019) and an SASP component (Coryell et al. 2021), known to activate downstream NF‐κB and p53 signaling pathways, either directly or indirectly via the activation of MAPK (Kwon et al. 2022; Sun et al. 2015) and PI3K (Barnes et al. 2019; W. Zhang et al. 2023) signaling pathways, which aligns with our KEGG findings. To illustrate the potential regulatory network, we integrated enrichment results with published signaling relationships into a schematic model (Figure 2K). This framework is intended to illustrate how senescent LECs secrete IL‐17A as part of their SASP repertoire, activating NF‐κB signaling that in turn reinforces SASP output and promotes EMT activation. Consistent with this framework, qRT‐PCR and Western blot confirmed significantly increased expression of IL‐17A and its receptor IL‐17RA in ARC‐PSC samples (Figure 2L–N).

Together, these results support a mechanistic model in which senescent LECs may contribute to an inflammatory microenvironment through SASP factors including IL‐17A, potentially linking cellular senescence to EMT activation in PSC.

### 3.3 Inflammatory Factors Are Elevated in the AqH of PSC

**FIGURE 3:**
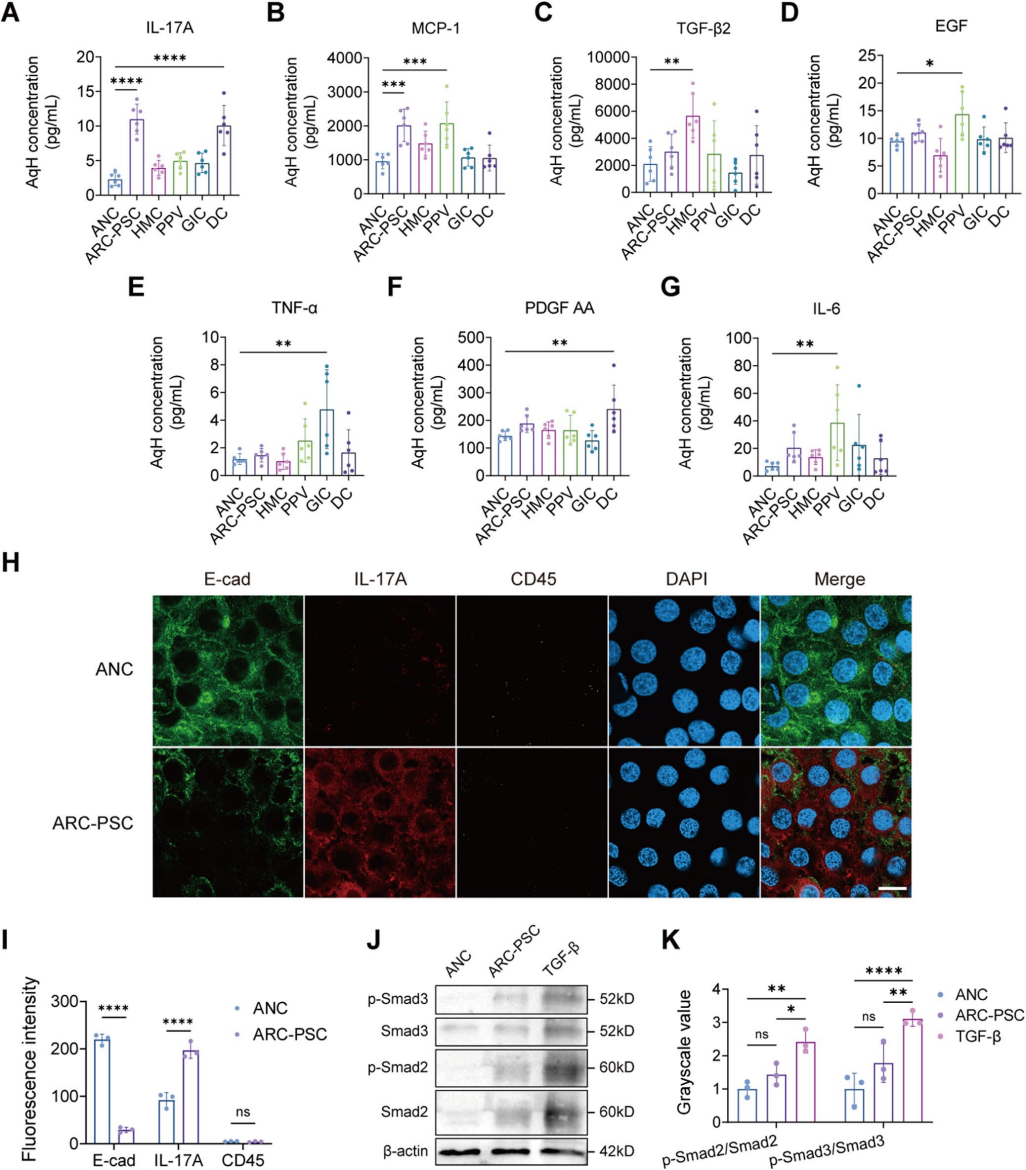
Inflammatory cytokine landscape and signaling features in posterior subcapsular cataracts (PSC). (A–G) Quantification of levels of IL‐17A (A), MCP‐1 (B), TGF‐β2 (C), EGF (D), TNF‐α (E), PDGF‐AA (F) and IL‐6 (G) in aqueous humor samples from PSC subgroups and ANC controls (*n* = 6 per group). (H–I) Representative IF images (H) and quantification (I) of anterior lens capsules stained for E‐cadherin (E‐cad, green), IL‐17A (red), and CD45 (yellow). DAPI (blue) was used for nuclear counterstaining. Scale bar: 10 μm. (J, K) Western blot (J) and densitometric analysis (K) of Smad2/3 and phosphorylated Smad2/3 (p‐Smad2/3) protein levels in the anterior lens capsules of age‐related nuclear cataract (ANC) and age‐related cataracts (ARC)‐PSC patients. TGF‐β‐treated samples were included as positive controls for canonical Smad activation. *n* = 3 biologically independent experiments. Data are presented as mean ± scanning electron microscopy (SEM). One‐way ANOVA was used for group comparisons. ns, not significant. *p < 0.05, **p < 0.01, ***p < 0.001, ****p < 0.0001.

To evaluate whether the molecular signatures detected in lens epithelium were mirrored in the surrounding ocular microenvironment, we profiled inflammatory factors in the AqH across different cataract subtypes. Consistent with transcriptomic findings, IL‐17A levels were significantly elevated in the ARC‐PSC group compared to age‐matched ANC controls, highlighting its potential role in this specific subtype. In contrast, IL‐17A was not increased in other PSC subtypes—including HMC, PPV, and GIC—except for a moderate elevation in the DC group (Figure 3A). These findings suggest a unique contribution of IL‐17A to ARC‐PSC.

Beyond IL‐17A, distinct subtype‐specific inflammatory patterns were evident. AqH from ARC‐PSC patients showed increased monocyte chemotactic protein (MCP)‐1 (Figure 3B), while HMC was characterized by elevated transforming growth factor (TGF)‐β2 (Figure 3C). PPV lenses demonstrated increased MCP‐1, epidermal growth factor (EGF), and interleukin (IL)‐6 (Figure 3B,D,G), whereas tumor necrosis factor (TNF)‐α and platelet‐derived growth factor (PDGF)‐AA were selectively upregulated in GIC and DC, respectively (Figure 3E,F).

To further determine the cellular source of IL‐17A in ARC‐PSC, we performed triple IF staining for IL‐17A, E‐cadherin, and the leukocyte marker CD45 in anterior capsules. In ANC samples, IL‐17A signal was minimal within the lens epithelium. By contrast, ARC‐PSC specimens exhibited broadly increased IL‐17A signals within the epithelial compartment, with IL‐17A detected in a subset of cells with residual E‐cadherin expression. CD45 staining was barely detectable across all groups (Figure 3H–I). Together, these findings indicate that IL‐17A in ARC‐PSC arises predominantly from LECs rather than infiltrating immune cells.

TGF‐β2, despite being a classical EMT inducer, was not significantly elevated in the AqH of ARC‐PSC (Figure 3C). Given that aqueous cytokine levels may not fully reflect local tissue signaling, we next examined whether canonical TGF‐β/Smad signaling was activated in the lens epithelium. Quantitative Western blot analysis demonstrated that phosphorylated Smad2/3 (p‐Smad2/3) levels in ARC‐PSC anterior capsules were comparable to ANC controls and markedly lower than those in TGF‐β‐treated positive‐control samples (Figure 3J–K). Together with the unchanged AqH TGF‐β2 levels, these data indicate that canonical TGF‐β/Smad signaling might not be the key contributing pathway in the ARC‐PSC pathology.

Taken together, these data reveal that PSC subtypes harbor distinct, SASP‐enriched inflammatory profiles in AqH. Among them, ARC‐PSC—a uniquely age‐related and inflammation‐minimal subtype—exhibits a characteristic increase in IL‐17A and MCP‐1, underscoring its utility as a model for studying senescence‐driven epithelial remodeling.

### 3.4 Senescent LECs Are Enriched in PSC and May Promote EMT and Disease Severity

**FIGURE 4:**
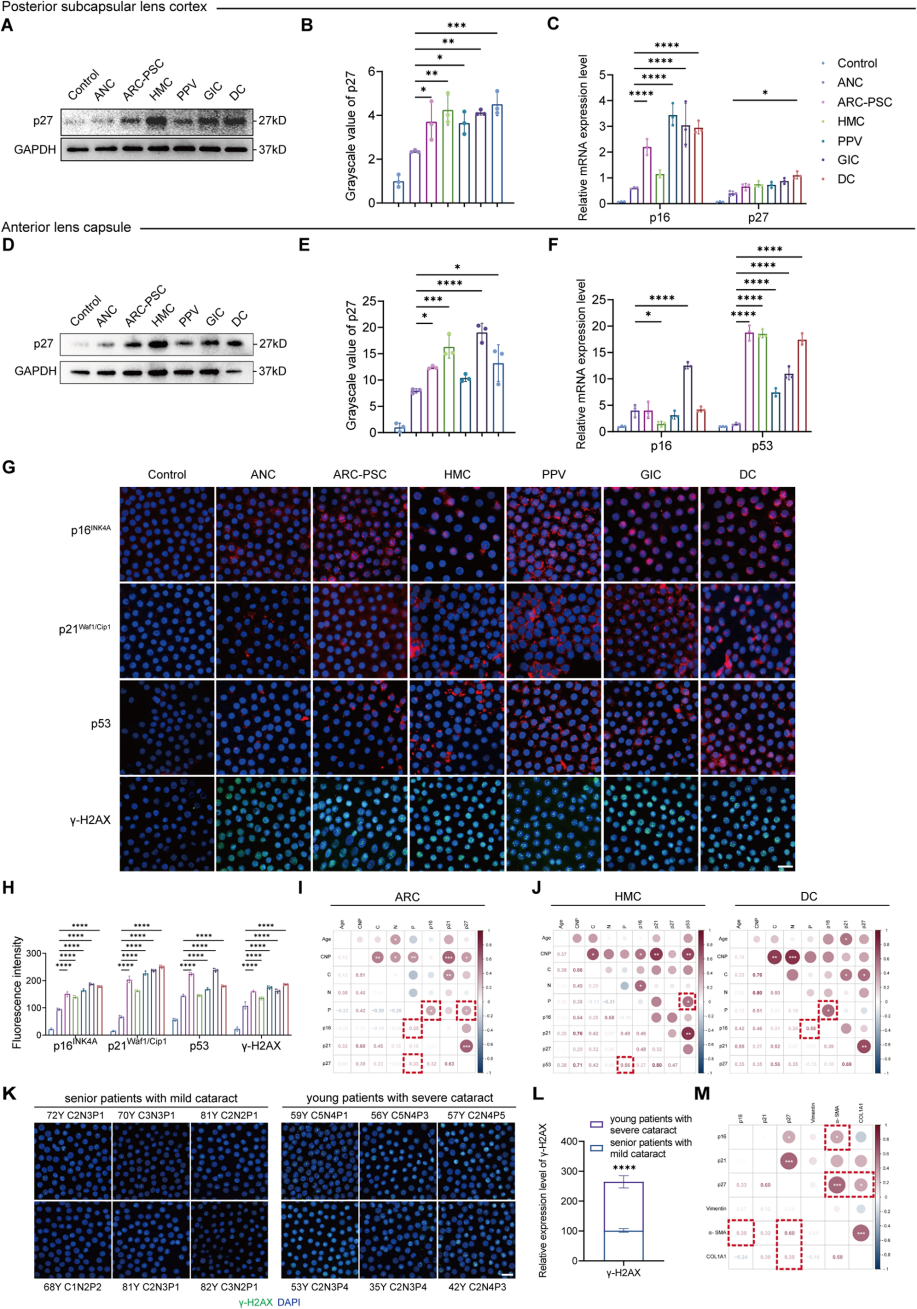
Cellular senescence in the posterior subcapsular lens cortex and anterior lens epithelium of posterior subcapsular cataracts (PSC) patients. (A, B) Western blot (A) and densitometric analysis (B) of p27^Kip1^ protein levels in the posterior subcapsular lens cortex. (C) Quantitative real‐time PCR (qRT‐PCR) analysis of p16^INK4A^ and p27^Kip1^ mRNA expression in posterior subcapsular lens cortex. (D, E) Western blot (D) and densitometric analysis (E) of p27^Kip1^ protein in anterior lens capsules. (F) qRT‐PCR analysis of p16^INK4A^ and p53 mRNA expression in anterior lens capsules. (G) Representative IF staining of anterior lens capsules for p16^INK4A^ (red), p21^Waf1/Cip1^ (red), p53 (red) and γ‐H2AX (green). DAPI (blue) labels cell nuclei. Scale bars: 20 μm. (H) Quantification of fluorescence intensity for p16^INK4A^, p21^Waf1/Cip1^, p53 and γ‐H2AX. (I, J) Spearman correlation analysis between cataract severity and expression of cellular senescence markers in age‐related cataract (ARC) (I), highly myopic cataract (HMC) and diabetic cataract (DC) (J). (K, L) Representative IF images (K) and intensity quantification (L) of γ‐H2AX (green) in ARC anterior lens capsule samples. DAPI (blue) labels cell nuclei. Scale bar: 20 μm. (M) Spearman correlation between EMT marker expression and senescence marker expression (mRNA level). Data are presented as mean ± scanning electron microscopy (SEM). One‐way ANOVA was used for multiple group comparisons; two‐tailed unpaired t‐test was used for two‐group comparisons in (L). Spearman correlation was used in (I, J, M). *n* = 3 biologically independent samples per group unless otherwise indicated. *p < 0.05, **p < 0.01, ***p < 0.001, ****p < 0.0001.

To confirm the presence of cellular senescence in PSC, we first examined canonical senescence markers in the posterior subcapsular cortex, where opacity originates in PSC. Western blot analysis revealed increased p27^Kip1^ levels in the opaque posterior subcapsular cortex of all PSC subtypes compared to ANC and normal lenses (Figure 4A,B). qRT‐PCR further showed significantly elevated p16^INK4A^ and p53 expression in the same region (Figure 4C).

We next assessed the anterior lens epithelium, the source of epithelial remodeling. Moderate but consistent increases were observed, with variability in p16^INK4A^ mRNA expression suggesting heterogeneous stages of senescence among epithelial cells (Figure 4D–F). IF staining further demonstrated elevated p16^INK4A^, p21^Waf1/Cip1^, p53 and γ‐H2AX in the lens epithelium of PSC samples (Figure 4G,H).

To assess clinical relevance, we examined associations between senescence markers and disease severity. Spearman correlation revealed significant positive correlations between p16^INK4A^ or p27^Kip1^ expression and posterior subcapsular opacity (Figure 4I). Subtype‐specific analysis indicated that p53 was most strongly associated with opacification in HMC, whereas p16^INK4A^ showed the highest correlation in DC (Figure 4J).

Notably, younger patients with severe PSC exhibited markedly higher γ‐H2AX levels than older individuals with milder disease (Figure 4K,L), suggesting that functional senescence may be more closely linked to disease progression than to chronological aging.

Finally, correlation analysis revealed strong positive associations between senescence and EMT marker expression across the patient cohort (Figure 4M). Together, these findings support a mechanistic model in which senescent LECs generate a SASP‐enriched microenvironment that sustains EMT and accelerates PSC progression.

### 3.5 IL‐17A Induces LEC Senescence via NF‐κB and Promotes Paracrine EMT Through Secretory Phenotype

**FIGURE 5:**
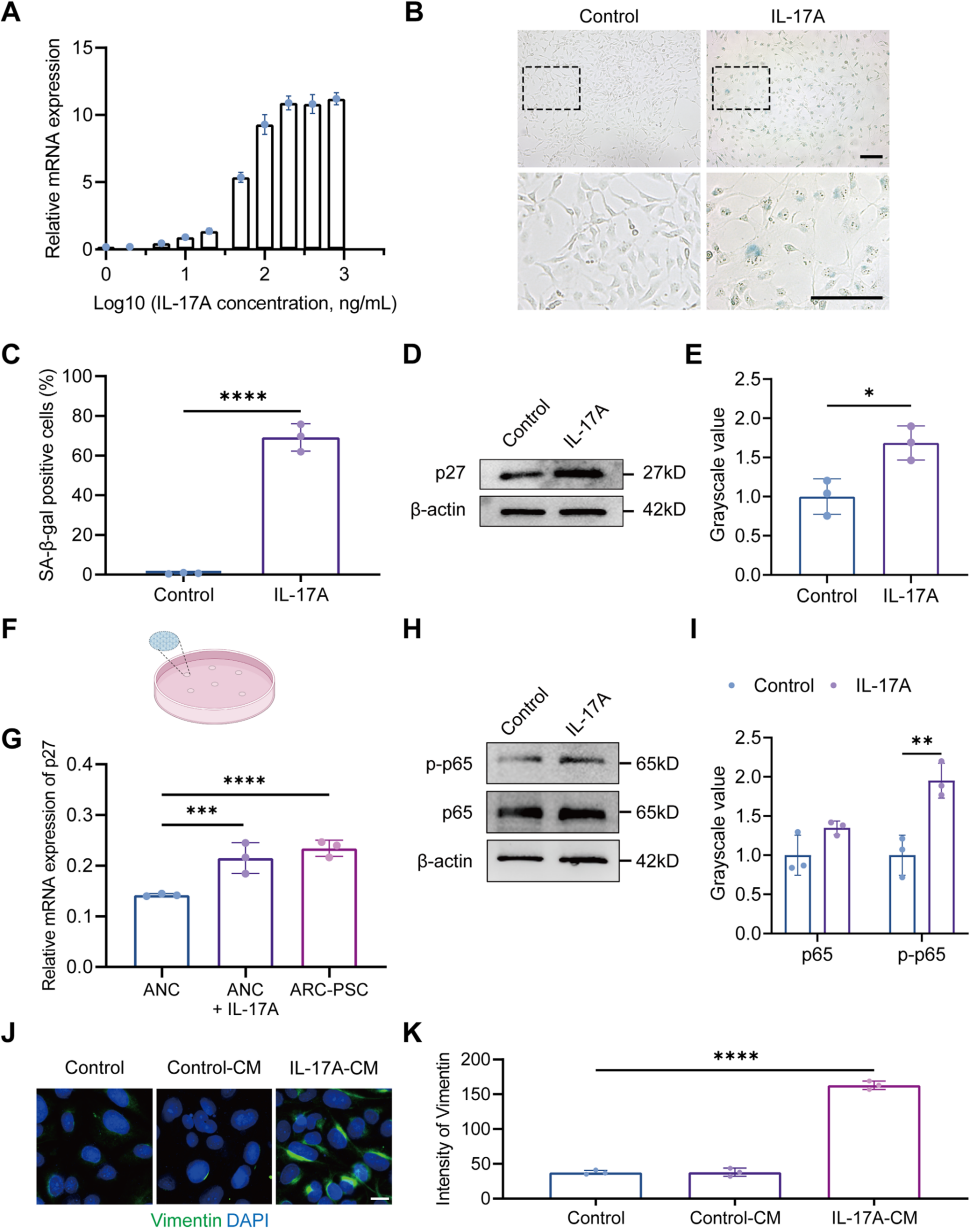
IL‐17A stimulation induces senescence and secondary epithelial–mesenchymal transition (EMT) features in cultured LECs and human anterior lens capsules. (A) Quantitative real‐time PCR (qRT‐PCR) analysis of MCP‐1 mRNA expression in cultured lens epithelial cells (LECs) treated with varying concentrations of recombinant human IL‐17A. X‐axis represents Log_10_‐transformed IL‐17A concentrations (ng/mL). (B) Representative images of SA‐β‐gal‐positive cells. Black dashed boxes indicate regions shown at higher magnification. Scale bars: 100 μm. (C) Quantification of SA‐β‐gal‐positive cells shown in panel B. (D, E) Western blot (D) and densitometric analysis (E) of p27^Kip1^ protein expression in LECs with or without IL‐17A treatment. (F) Schematic of ex vivo human anterior lens capsule culture system. Diagram created with Figdraw. (G) qRT‐PCR analysis of p27^Kip1^ mRNA expression in cultured anterior lens capsules from age‐related cataracts (ARC)‐posterior subcapsular cataracts (PSC) and age‐related nuclear cataract (ANC) patients, treated or untreated with IL‐17A (200 ng/mL). (H, I) Western blot (H) and densitometric analysis (I) illustrating the protein expression levels of p65 and phospho‐p65 in different groups. (J, K) Representative images of IF staining (J) and intensity analysis (K) of LECs treated with conditioned medium (CM) demonstrating positivity of Vimentin (green). DAPI staining (blue) labels the cell nuclei. Scale bar: 20 μm. Data are shown as mean ± SEM. *n* = 3 biologically independent samples per group. One‐way ANOVA was used for multiple group comparisons. *p < 0.05, **p < 0.01, ***p < 0.001, ****p < 0.0001.

Because IL‐17A emerged from transcriptomic and AqH analyses as a candidate SASP mediator, we next explored whether IL‐17A could induce senescence in LECs and promote paracrine EMT through its secretory phenotype, using a series of in vitro and ex vivo experiments. Recombinant IL‐17A treatment of cultured human LECs induced a dose‐dependent increase in MCP‐1 expression, plateauing at 200 ng/mL (Figure 5A), consistent with prior observations (Liao et al. 2021). IL‐17A exposure also increased the proportion of SA‐β‐gal‐positive senescent cells (Figure 5B,C) and elevated p27^Kip1^ protein levels (Figure 5D,E), indicating activation of senescence‐associated pathways.

To examine these effects in a more physiologically relevant context, we applied IL‐17A to ex vivo cultured anterior lens capsules from ANC patients (Figure 5F). qRT‐PCR revealed increased expression of p27^Kip1^ in treated explants, supporting IL‐17A's ability to promote senescence in native lens epithelial tissues (Figure 5G).

We next asked whether IL‐17A‐induced senescence was mediated through the NF‐κB pathway. Western blot analysis showed increased phosphorylation of NF‐κB p65 following IL‐17A exposure (Figure 5H,I), consistent with pathway activation. These findings place NF‐κB as a key mediator linking IL‐17A signaling to senescence onset in LECs.

To explore whether IL‐17A‐induced senescent LECs could promote EMT in a paracrine manner, IL‐17A‐treated cells were washed and cultured in IL‐17A‐free medium, and their conditioned supernatant was applied to untreated LECs. IF analysis showed increased Vimentin expression in these recipient cells (Figure 5J,K), suggesting that factors secreted by senescent cells—presumably SASP components—can induce EMT‐like changes in neighboring cells.

Together, these findings indicate that IL‐17A induces senescence in LECs via NF‐κB activation, and the secretory phenotype of senescent cells subsequently drives secondary EMT. This provides a functional link between LEC senescence and pathological epithelial remodeling in PSC. Notably, these results also reinforce the notion that senescent LECs function primarily as signaling sources, rather than direct participants, in EMT remodeling.

### 3.6 Targeting Senescent Cells or IL‐17A/NF‐κB Signaling Suppresses EMT

Having shown that IL‐17A could induce cellular senescence in LECs, we next examined whether senescent LECs themselves could initiate EMT in surrounding cells through paracrine signaling. Given the established role of oxidative stress in cataractogenesis (Spector 1995), we established in vitro models of premature senescence in LECs using both exogenous (H_2_O_2_) and endogenous (glucose oxidase, GOx) oxidative stressors (Figure S2A,B). As expected, these stress conditions induced premature senescence (Figure S2C–F). Conditioned medium (CM) from these senescent LECs robustly induced Vimentin expression in untreated cells (Figure S2G,H), consistent with a paracrine mechanism whereby senescent cells promote EMT in neighboring epithelial cells. In these models, IL‐17RA mRNA displayed a mild, non‐significant increase (Figure S2I,J).

To dissect therapeutic avenues that could interrupt this senescence–EMT axis, we applied two complementary strategies: clearance of senescent cells with Procyanidin C1 (PCC1) (Xu et al. 2021), and inhibition of IL‐17A with the clinically validated antibody Secukinumab (Figure S3A–C).

**FIGURE 6:**
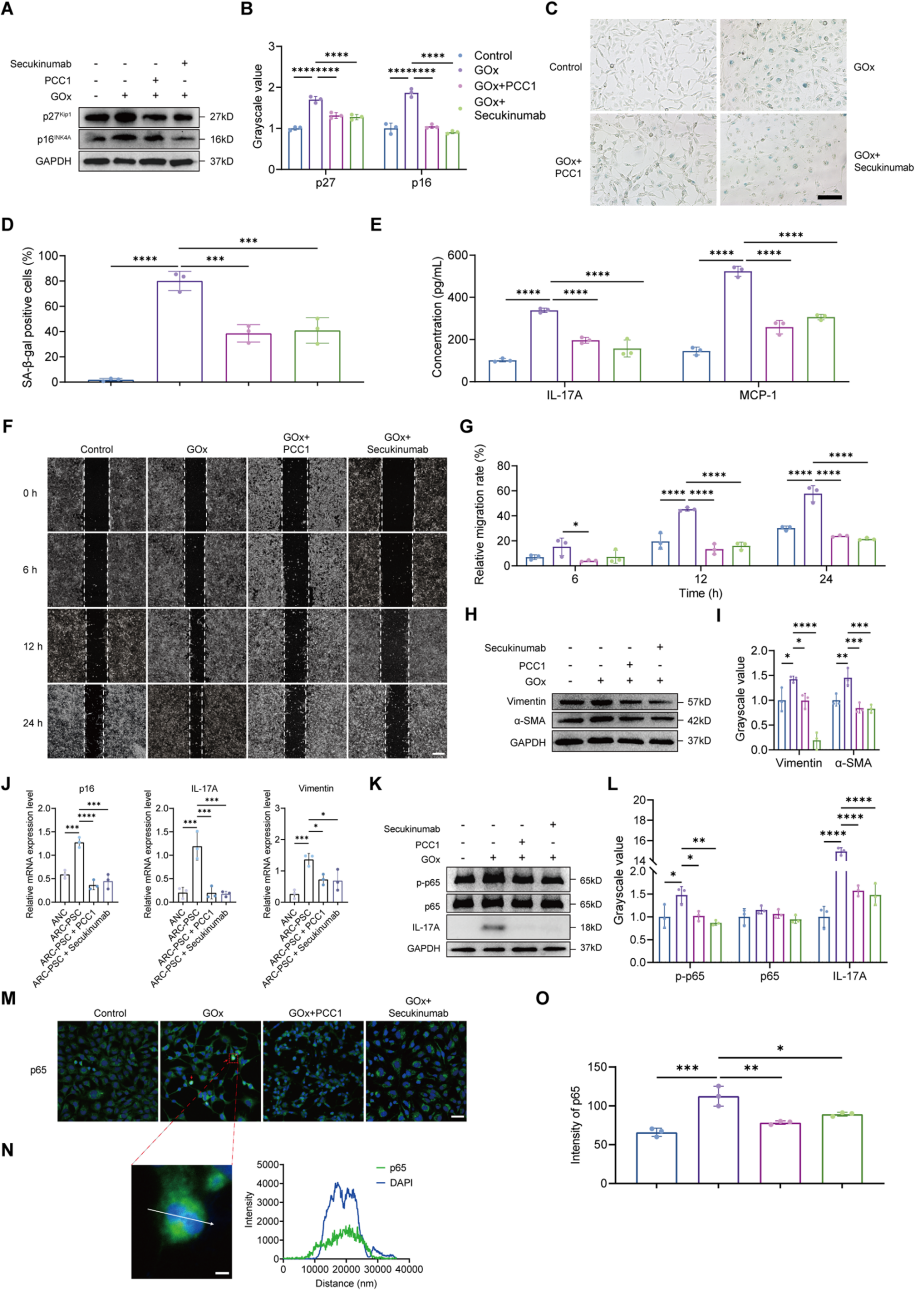
PCC1 and IL‐17A blockade reduce inflammaging and epithelial–mesenchymal transition (EMT) features via inhibition of the IL‐17A/NF‐κB signaling pathway. (A, B) Western blot (A) and densitometric analysis (B) of p16^INK4A^ and p27^Kip1^ protein expression under different treatment conditions. (C, D) Representative images (C) and quantification (D) of SA‐β‐gal positive cells. Scale bar: 50 μm. (E) ELISA quantification of IL‐17A and MCP‐1 protein levels in culture supernatants of LECs treated with GOx (30 mU/mL), PCC1 (20 μM), or Secukinumab (70 nM), alone or in combination. (F, G) Representative wound healing images (F) and migration rate analysis (G) showing the migration ability of cultured cells. Scale bar: 50 μm. (H, I) Western blot (H) and densitometric analysis (I) of EMT markers Vimentin and α‐SMA in different groups. (J) Quantitative real‐time PCR (qRT‐PCR) analysis of p16^INK4A^, IL‐17A, and Vimentin mRNA levels in ex vivo cultured anterior lens capsules from age‐related cataracts (ARC)‐posterior subcapsular cataracts (PSC) and age‐related nuclear cataract (ANC) patients untreated or treated with PCC1 (20 μM) and Secukinumab (70 nM). (K, L) Western blot (K) and densitometric analysis (L) of IL‐17A, total p65, and phosphorylated p65 protein levels in different groups. (M) Representative IF images of cells demonstrating positivity of p65 (green). DAPI (blue) labels cell nuclei. Red arrows indicate the nuclear translocation of p65. Scale bar: 50 μm. (N) Representative IF images of p65 (green) nuclear translocation. DAPI (blue) labels cell nuclei. Line diagram illustrating the colocalization of p65 with the nucleus along the path indicated by the white arrow. Scale bar: 5 μm. (O) Quantification of p65 fluorescence intensity in LECs. Data are presented as mean ± scanning electron microscopy (SEM). *n* = 3 biologically independent samples per group. One‐way ANOVA was used for all group comparisons. *p < 0.05, **p < 0.01, ***p < 0.001, ****p < 0.0001.

Both treatments effectively attenuated cellular senescence and its associated secretory phenotype. Secukinumab and PCC1 reduced senescence markers, including p16^INK4A^ and p27^Kip1^ (Figure 6A,B, Figure S4A,B), and decreased the proportion of SA‐β‐gal‐positive cells (Figure 6C,D), indicating attenuation of senescent phenotypes.

In parallel, both treatments reduced intracellular levels of key SASP‐related cytokines, such as IL‐6 and TNF‐α (Figure S5A), and also lowered secreted IL‐17A and MCP‐1 in culture supernatants (Figure 6E, Figure S4C), indicating suppression of SASP output at both intracellular and extracellular levels.

Both interventions also mitigated EMT‐like changes. Cell migration was reduced in wound healing assays (Figure 6F,G, Figure S4D,E), and EMT markers including Vimentin and α‐SMA were downregulated (Figure 6H,I, Figure S4F,G). In ex vivo‐cultured human anterior lens capsules, Secukinumab and PCC1 reduced p16^INK4A^, IL‐17A, and Vimentin expression, reinforcing their modulatory effects in a physiologically relevant model (Figure 6J).

Mechanistically, both interventions converged on NF‐κB signaling. Secukinumab, as expected, blocked IL‐17A‐mediated NF‐κB activation, as shown by reduced p65 phosphorylation (Figure 6K,L, Figure S6A,B). PCC1 also reduced IL‐17A expression, suggesting that senescent cells contribute to sustaining this signaling axis. These observations support a senescence‐centered feedback loop, in which senescent LECs secrete IL‐17A as part of their SASP, activating NF‐κB signaling that amplifies SASP output and reinforces both senescence and EMT in neighboring cells. IF analysis supported this model, showing decreased nuclear translocation and intensity of p65 following either intervention (Figure 6M–O).

Together, these results position cellular senescence as an upstream pathological hub that maintains EMT through SASP‐mediated paracrine signaling. Disrupting either the accumulation of senescent cells or the IL‐17A/NF‐κB amplification axis effectively attenuates EMT‐like phenotypes, providing mechanistic insight and suggesting therapeutic potential for targeting ARC‐PSC.

## 4 Discussion

PSC are clinically important for their early visual impact and surgical risks, yet their underlying mechanisms remain poorly defined. Our analysis of clinically stratified human samples revealed EMT as a recurrent feature across PSC subtypes, with ARC‐PSC showing robust EMT activity in the absence of external triggers. These observations establish epithelial remodeling—beyond protein aggregation alone—as a key mechanism of PSC pathogenesis. In this context, ARC‐PSC serves as a tractable human model for studying intrinsic, age‐related processes. Extending these observations, we identify cellular senescence and its associated secretory program as key drivers of epithelial remodeling, thereby defining a senescence–SASP–EMT signaling axis in PSC.

The classical model of cataractogenesis centers on crystallin modifications and aggregation under oxidative and metabolic stress (Moreau and King 2012; Weinberg et al. 2022), which account for much of the pathology in nuclear and cortical cataracts. However, this protein‐centric framework does not adequately explain the spatial distinctiveness and rapid progression of PSC. While EMT has been previously implicated in complicated cataracts such as HMC (De Piano et al. 2023), GIC (Yoo et al. 2023), and DC (T.‐T. Wu et al. 2020), our findings identify epithelial remodeling—marked by EMT—as a complementary pathological mechanism, particularly evident in ARC‐PSC. Taken together, the integration of protein instability and epithelial plasticity offers a more complete explanation of cataract heterogeneity. Notably, in ARC‐PSC, anterior LECs display only subtle morphological alterations despite molecular evidence of EMT reprogramming. This restrained phenotype likely reflects anatomical constraints imposed by the intact anterior capsule and spatial separation from the posterior subcapsular lesion site. Thus, EMT marker expression in the anterior capsule may represent early and/or partial remodeling rather than fully executed morphological EMT. Within this framework, the emergence of EMT in ARC‐PSC—independent of trauma, surgery, or overt inflammation—raises a central mechanistic question: what endogenous processes drive epithelial remodeling in the aging lens?

To explore potential endogenous drivers of EMT in ARC‐PSC, we focused on cellular senescence and examined its relationship with EMT within lens tissues. Senescence markers were detected both in the posterior subcapsular cortex, where opacity originates, and in the anterior lens epithelium, the site of viable LECs. Functionally, senescence is not merely a passive marker of aging, but actively reshapes the local tissue microenvironment through SASP. SASP factors can propagate senescence and induce EMT‐like changes in adjacent epithelial cells, thereby forming a self‐reinforcing paracrine loop (Franceschi et al. 2018; X. Li et al. 2023). This non‐cell‐autonomous effect explains why senescent LECs themselves do not undergo EMT, but instead drive secondary epithelial remodeling through paracrine signaling.

Taken together, these findings support a signaling‐based rather than fate‐based model of lens remodeling, wherein cumulative senescence—not direct epithelial transdifferentiation—triggers EMT. Importantly, our findings suggest that the extent of SASP activity may shape whether senescence‐associated changes progress to overt EMT. Although ANC and ARC‐PSC share senescence‐associated transcriptional changes, as reflected by pathway overlap in the Venn analysis (Figure 2A), only ARC‐PSC exhibits EMT‐related remodeling. This divergence is associated with markedly higher SASP activity in ARC‐PSC, as evidenced by the enrichment of SASP‐related DEGs and their validation in anterior lens capsules (Figure 2I,J).

Among the SASP cytokines enriched in ARC‐PSC, IL‐17A emerged as a representative mediator linking senescence to EMT. While other SASP factors such as IL‐6, MCP‐1, and TNF‐α were also elevated, IL‐17A stood out because it was uniquely consistent across transcriptomic, proteomic, and AqH profiling, was mechanistically linked to NF‐κB activation (Liao et al. 2021; Solá et al. 2023; Wang et al. 2019), and is therapeutically tractable due to the availability of clinically validated inhibitors. Functional assays further demonstrated that IL‐17A can induce senescence in LECs and promote EMT in neighboring cells through paracrine signaling, whereas neutralization of IL‐17A reduced these phenotypes in parallel with diminished NF‐κB activation, highlighting its role as an effector rather than a mere correlate of SASP‐mediated epithelial remodeling. To clarify its cellular origin in ARC‐PSC, we performed triple IF staining for IL‐17A, E‐cadherin, and CD45 in anterior capsule whole mounts. CD45^+^ immune cells were barely detectable, whereas IL‐17A signals were predominantly localized within the epithelial compartment and spatially associated with regions marked by residual E‐cadherin expression. Although typically considered immune‐derived, IL‐17A has also been reported from epithelial cells under stress conditions (Z. J. Li et al. 2016; Loverre et al. 2011; M. Wu et al. 2021), supporting the plausibility of an epithelial origin of IL‐17A in ARC‐PSC. Consistent with this, IL‐17RA expression remained largely unchanged in senescence models in vitro but was elevated in capsule samples, suggesting that chronic in vivo stress may enhance receptor sensitivity to IL‐17A signaling (Sarma et al. 2009).

Building on the identification of IL‐17A as a representative SASP effector, we next asked whether targeting the upstream source of SASP could more effectively disrupt the senescence–SASP–EMT circuit. To this end, we employed PCC1, a flavonoid‐based senolytic compound previously shown to selectively eliminate senescent cells (Xu et al. 2021). Unlike cytokine blockade, this approach acts directly on the cellular origin of SASP (Y. Liu et al. 2024). PCC1 treatment reduced IL‐17A output, suppressed NF‐κB activation, and attenuated EMT‐associated changes in both cultured LECs and ex vivo human lens capsules, without exerting broad cytotoxic effects on non‐senescent cells. These findings confirm that senescent LECs function primarily as upstream signal sources, with NF‐κB serving as an amplifier of SASP output rather than a direct EMT trigger. By eliminating senescent cells, PCC1 interrupts this feed‐forward loop and halts pathological epithelial remodeling. Beyond mechanistic validation, these results also highlight the translational potential of senescence‐targeted interventions for ARC‐PSC and, more broadly, for epithelial degenerative disorders in which senescence–EMT coupling has been implicated.

Although ARC‐PSC was used as our primary model, this reflects its advantage as a minimally confounded, intrinsically aging‐driven setting rather than a subtype‐specific mechanism. Across PSC subtypes with diverse clinical triggers, senescence‐associated changes and SASP mediators were consistently detectable alongside EMT, suggesting convergence on a shared senescence–SASP–EMT program. Notably, the cellular sources of SASP factors are likely context‐dependent across subtypes (e.g., inflammatory, metabolic, or surgical stress), even though the downstream epithelial response in the lens is largely shared. Thus, ARC‐PSC provides the clearest window into a generalizable aging‐encoded epithelial plasticity program that underlies PSC more broadly.

In summary, our study positions cellular senescence as a key upstream process driving pathological epithelial remodeling in PSC. Senescent LECs act as signaling sources, releasing SASP factors such as IL‐17A that activate NF‐κB and promote EMT in neighboring cells. This senescence–SASP–EMT loop is supported by molecular, morphological, and functional evidence in a clinically stratified human cohort. Unlike conventional animal models, which rarely reproduce the subcapsular phenotype of ARC‐PSC, our human‐based system enables direct mechanistic dissection of age‐related epithelial remodeling in situ. Within this framework, ARC‐PSC provides not only a clinically relevant cataract subtype but also a naturally occurring human context for studying senescence‐driven epithelial plasticity. By establishing this framework, our work delivers human‐specific mechanistic insights into how chronic senescence reshapes epithelial tissues within the aging lens.

## Author Contributions

Conceptualization: M.W., Y.Q., and R.J. Methodology: Y.Q. and R.J.; Y.N., and Z.R. conducted experiments; Y.M., Y.Z., and L.L. conducted data analysis; R.D. conducted and interpreted RNA‐seq analysis. Visualization: Y.C. and L.Q. Funding acquisition: M.W. and Y.Q. Writing – original draft: Y.N. Writing – review and editing: M.W., Y.Q., and R.J.

## Funding

The work is funded by Natural Science Foundation of Guangdong Province, China (Grant No. 2025A1515012676); Science and Technology Program of Guangzhou, China (Grant No. SL2024A03J00523); National Natural Science Foundation of China (Grant No. 82501263, Grant No. 81970783); Guangzhou basic and applied basic research foundation, China (Grant No. 2024A04J4473).

## Ethics Statement

The study protocol was approved by the Ethical Review Committee of the Zhongshan Ophthalmic Center (Approval No. 2021KYPJ168‐2), and all procedures adhered to the tenets of the Declaration of Helsinki. Written informed consent was obtained from all participants prior to sample collection.

## Conflicts of Interest

The authors declare no conflicts of interest.

## Supporting information


**Figure S1:** Details of differentially expressed genes (DEGs) identified through bulk RNA‐seq bulk analysis among control, age‐related nuclear cataract (ANC) and age‐related cataracts (ARC)‐posterior subcapsular cataracts (PSC) groups. (A) Venn diagram displaying the number of DEGs shared among control, ANC and ARC‐PSC groups. (B, C) Volcano plots showing DEGs between the following comparisons: ARC‐PSC versus control (B) and ARC‐PSC versus ANC (C). The plots are displayed on a −log_10_ scale for statistical significance (*p*‐value) and a log_2_ scale for fold change. Significantly up‐ and downregulated genes (*p*‐value < 0.05 & absolute fold change > 2) are highlighted in red and blue respectively as indicated. Non‐significantly regulated genes are shown in gray.
**Figure S2:** Pro‐inflammatory phenotype, cellular senescence and epithelial–mesenchymal transition (EMT) changes in cell models. (A, B) Fitting curves illustrating the effects of different concentrations of H_2_O_2_ (A) and GOx (B) on cell viability. The IC50 value (half‐maximal inhibitory concentration) indicate the concentration at which 50% of cell viability is inhibited. (C) Representative western blot images illustrating the protein expression levels of senescence markers in control and H_2_O_2_‐treated groups. (D) ELISA assay showing the supernatant level of IL‐17A and MCP‐1 in control and H_2_O_2_‐treated groups. (E) Representative western blot images illustrating the protein expression levels of senescence markers in control and GOx‐treated groups. (F) ELISA assay showing the supernatant level of IL‐17A and MCP‐1 in control and GOx‐treated groups. (G, H) Representative images of immunofluorescence staining (G) and intensity analysis (H) of lens epithelial cells (LECs) treated with conditioned medium (CM) demonstrating positivity of Vimentin (green). DAPI staining (blue) labels the cell nuclei. Scale bars: 20 μm. (I) Quantitative real‐time PCR (qRT‐PCR) analysis of IL‐17RA mRNA expression in control and H_2_O_2_‐treated groups. (J) qRT‐PCR analysis of IL‐17RA mRNA expression in control and GOx‐treated groups. Data are presented as the mean ± scanning electron microscopy (SEM). One‐way ANOVA was used for multiple group comparisons; two‐tailed unpaired *t*‐test was used for two‐group comparisons in (I, J). ns, not significant. ****p < 0.0001.
**Figure S3:** Dose–response curves of cell viability at varying drug concentrations. (A, B) Fitting curves illustrating the effects of different concentrations of Secukinumab (A) and PCC1 (B) on cell viability. The IC50 value (half‐maximal inhibitory concentration) indicate the concentration at which 50% of cell viability is inhibited. (C) Bar chart showing cell viability of normal and senescent cells under different concentrations of PCC1. Data are presented as the mean ± S scanning electron microscopy (SEM). All analyses were conducted using a one‐way ANOVA test. ns, not significant. **p < 0.01, ***p < 0.001, ****p < 0.0001.
**Figure S4:** PCC1 alleviated premature senescence and EMT in H_2_O_2_‐treated lens epithelial cells (LECs). (A, B) Representative western blot images (A) and densitometric analysis (B) illustrating the protein expression levels of senescence markers in different groups, normalized to GAPDH in each lane, with the average levels in the control group set as 1. (C) ELISA assay showing the supernatant level of IL‐17A and MCP‐1 untreated or treated with H_2_O_2_ (200 μM), PCC1 (20 μM) and Secukinumab (70 nM). (D, E) Representative wound healing images (D) and migration rate analysis (E) showing the migration ability of LECs untreated or treated with H_2_O_2_ (200 μM), PCC1 (20 μM) and Secukinumab (70 nM). Scale bar: 50 μm. (F, G) Representative western blot images (F) and densitometric analysis (G) illustrating the protein expression levels of EMT‐related markers in different groups. Data are presented as the mean ± SEM. All analyses were conducted using a one‐way ANOVA test. *p < 0.05, **p < 0.01, ***p < 0.001, ****p < 0.0001.
**Figure S5:** PCC1 and Secukinumab suppress GOx‐induced pro‐inflammatory cytokines in lens epithelial cells (LECs). (A) Quantitative real‐time PCR (qRT‐PCR) analysis of IL‐6 and TNF‐α mRNA levels in cultured cells from different groups. Data are presented as the mean ± scanning electron microscopy (SEM). All analyses were conducted using a one‐way ANOVA test. ****p < 0.0001.
**Figure S6:** PCC1 alleviated inflammaging‐induced epithelial–mesenchymal transition (EMT) in H_2_O_2_‐treated cells via IL‐17A/NF‐κB signaling pathway. (A, B) Representative western blot images (A) and densitometric analysis (B) illustrating the protein expression levels of IL‐17A, p65 and p‐p65 in different groups. Data are presented as the mean ± scanning electron microscopy (SEM). All analyses were conducted using a one‐way ANOVA test. *p < 0.05, **p < 0.01, ***p < 0.001, ****p < 0.0001.
**Table S1:** Basic details of clear lens donors.
**Table S2:** Reagents, kits, and instruments used in this study.
**Table S3:** Antibodies used in the experiments.
**Table S4:** Primers for quantitative real‐time PCR (qRT‐PCR) used in the experiments.

## Data Availability

The data that support the findings of this study are available from the corresponding author upon reasonable request.
