# Supplementary material for: Senescence‐Driven IL‐17A Inflammatory Circuit Promotes Epithelial–Mesenchymal Transition (EMT) and Progression in Age‐Related Posterior Subcapsular Cataracts
Source: Aging Cell. 2026 Mar 24;25(4):e70456. doi: 10.1111/acel.70456 (PMC13092507; doi:10.1111/acel.70456)
Supplement: Supplementary file 1 — Figure S1: Details of differentially expressed genes (DEGs) identified through bulk RNA‐seq bulk analysis among control, age‐related nuclear cataract (ANC) and age‐related cataracts (ARC)‐posterior subcapsular cataracts (PSC) groups. (A) Venn diagram displaying the number of DEGs shared among control, ANC and ARC‐PSC groups. (B, C) Volcano plots showing DEGs between the following comparisons: ARC‐PSC versus control (B) and ARC‐PSC versus ANC (C). The plots are displayed on a −log10 scale for statistical significance (p‐value) and a log2 scale for fold change. Significantly up‐ and downregulated genes (p‐value < 0.05 & absolute fold change > 2) are highlighted in red and blue respectively as indicated. Non‐significantly regulated genes are shown in gray. Figure S2: Pro‐inflammatory phenotype, cellular senescence and epithelial–mesenchymal transition (EMT) changes in cell models. (A, B) Fitting curves illustrating the effects of different concentrations of H2O2 (A) and GOx (B) on cell viability. The IC50 value (half‐maximal inhibitory concentration) indicate the concentration at which 50% of cell viability is inhibited. (C) Representative western blot images illustrating the protein expression levels of senescence markers in control and H2O2‐treated groups. (D) ELISA assay showing the supernatant level of IL‐17A and MCP‐1 in control and H2O2‐treated groups. (E) Representative western blot images illustrating the protein expression levels of senescence markers in control and GOx‐treated groups. (F) ELISA assay showing the supernatant level of IL‐17A and MCP‐1 in control and GOx‐treated groups. (G, H) Representative images of immunofluorescence staining (G) and intensity analysis (H) of lens epithelial cells (LECs) treated with conditioned medium (CM) demonstrating positivity of Vimentin (green). DAPI staining (blue) labels the cell nuclei. Scale bars: 20 μm. (I) Quantitative real‐time PCR (qRT‐PCR) analysis of IL‐17RA mRNA expression in control and H2O2‐treated groups. [file ACEL-25-e70456-s001.docx]

Supporting Information

**Supplementary Figures**


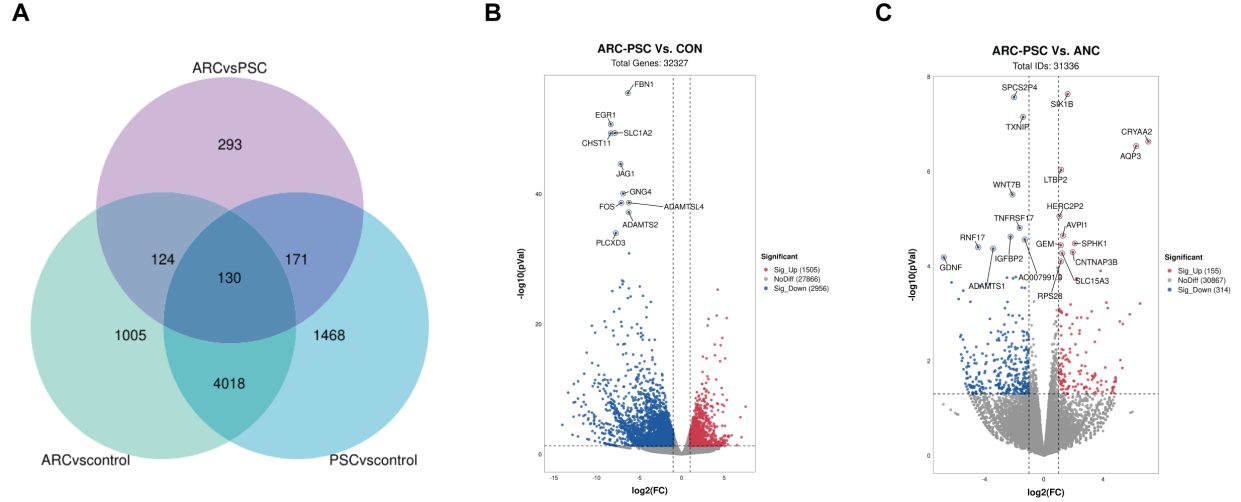


**Figure S1. Details of DEGs identified through bulk RNA-seq bulk analysis among control, ANC and ARC-PSC groups. (A)** Venn diagram displaying the number of DEGs shared among control, ANC and ARC-PSC groups. **(B-C)** Volcano plots showing DEGs between the following comparisons: ARC-PSC vs. control **(B)** and ARC-PSC vs. ANC **(C)**. The plots are displayed on a -log10 scale for statistical significance (*p*-value) and a log2 scale for fold change. Significantly up- and downregulated genes (*p*-value < 0.05 & absolute fold change > 2) are highlighted in red and blue respectively as indicated. Non-significantly regulated genes are shown in gray.


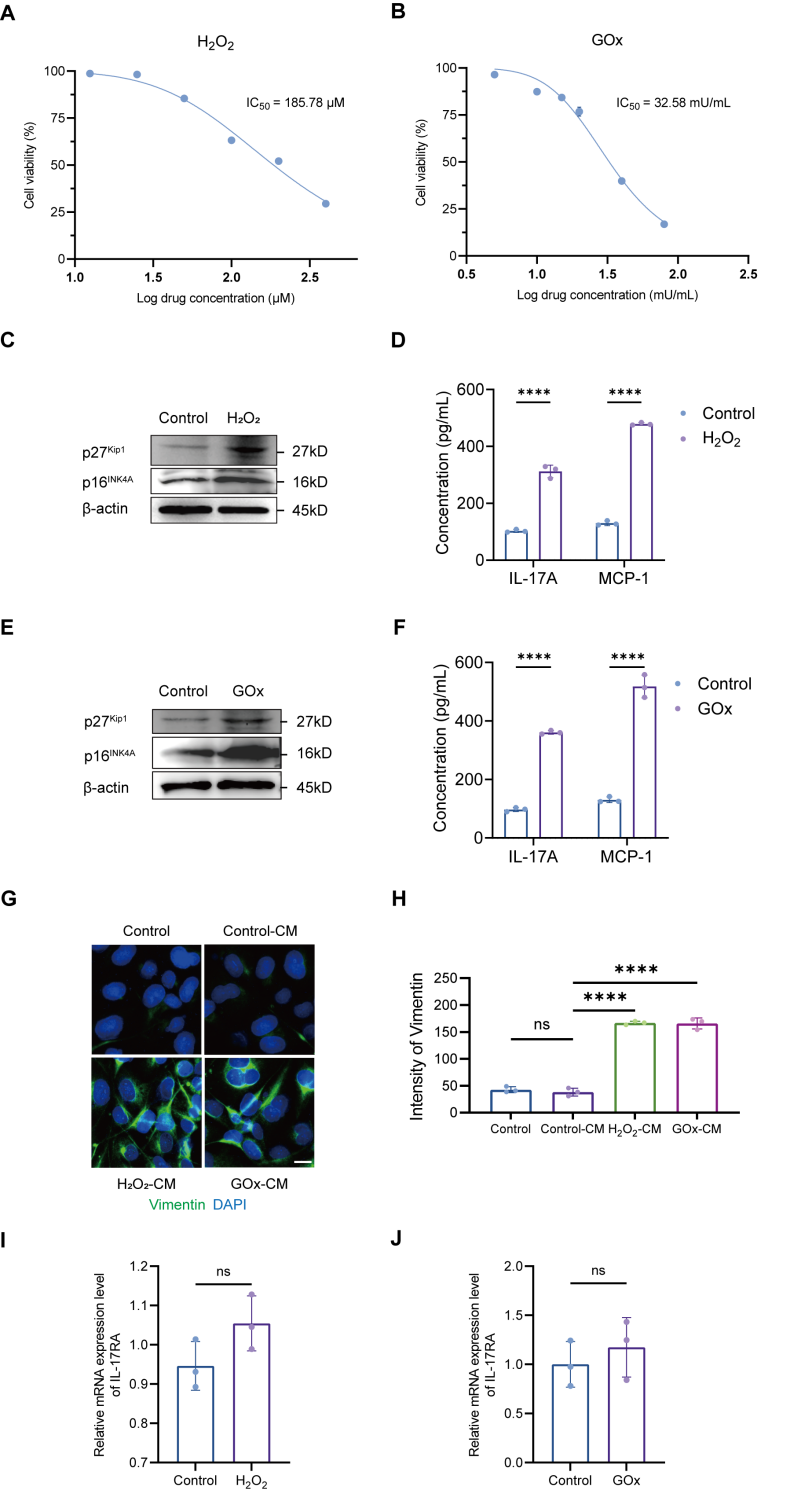


**Figure S2. Pro-inflammatory phenotype, cellular senescence and EMT changes in cell models. (A-B)** Fitting curves illustrating the effects of different concentrations of H_2_O_2_ **(A)** and GOx **(B)** on cell viability. The IC_50_ value (half-maximal inhibitory concentration) indicate the concentration at which 50% of cell viability is inhibited. **(C)** Representative western blot images illustrating the protein expression levels of senescence markers in control and H_2_O_2_-treated groups. **(D)** ELISA assay showing the supernatant level of IL-17A and MCP-1 in control and H_2_O_2_-treated groups. **(E)** Representative western blot images illustrating the protein expression levels of senescence markers in control and GOx-treated groups. **(F)** ELISA assay showing the supernatant level of IL-17A and MCP-1 in control and GOx-treated groups. **(G-H)** Representative images of immunofluorescence staining **(G)** and intensity analysis **(H)** of LECs treated with conditioned medium (CM) demonstrating positivity of Vimentin (green). DAPI staining (blue) labels the cell nuclei. Scale bars: 20μm. **(I)** qRT-PCR analysis of IL-17RA mRNA expression in control and H_2_O_2_-treated groups. **(J)** qRT-PCR analysis of IL-17RA mRNA expression in control and GOx-treated groups. Data are presented as the mean ± SEM. One-way ANOVA was used for multiple group comparisons; two-tailed unpaired t test was used for two-group comparisons in (I, J). ns, not significant. *****p* < 0.0001.


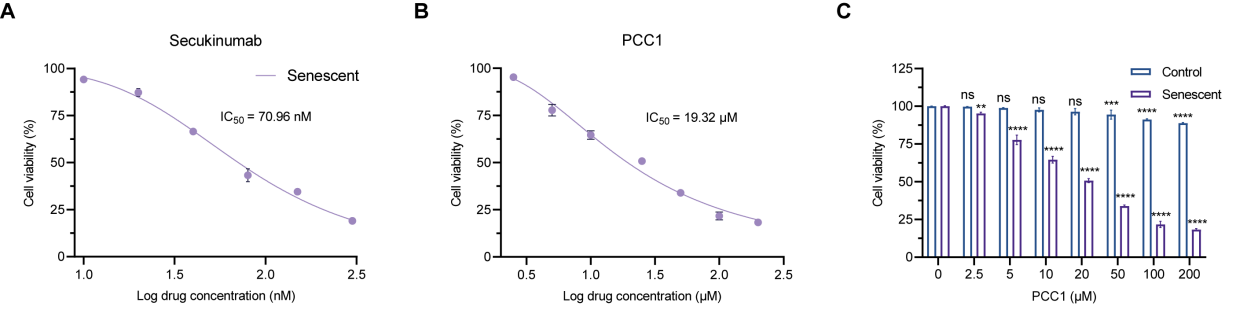


**Figure S3. Dose-response curves of cell viability at varying drug concentrations. (A-B)** Fitting curves illustrating the effects of different concentrations of Secukinumab **(A)** and PCC1 **(B)** on cell viability. The IC_50_ value (half-maximal inhibitory concentration) indicate the concentration at which 50% of cell viability is inhibited. **(C)** Bar chart showing cell viability of normal and senescent cells under different concentrations of PCC1. Data are presented as the mean ± SEM. All analyses were conducted using a one-way ANOVA test. ns, not significant. ***p* < 0.01, ****p* < 0.001, *****p* < 0.0001.


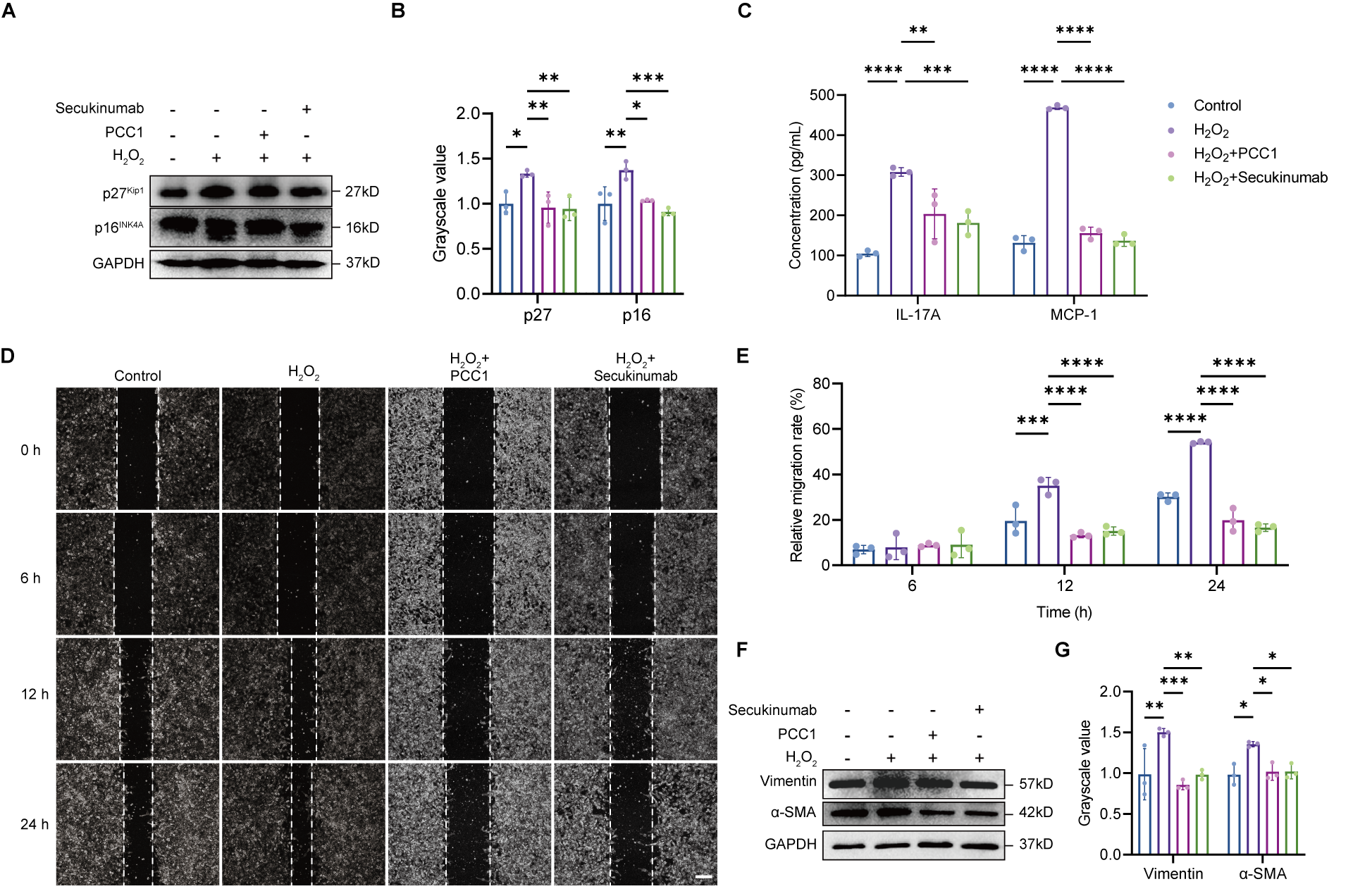


**Figure S4. PCC1 alleviated premature senescence and EMT in H_2_O_2_-treated LECs. (A-B)** Representative western blot images **(A)** and densitometric analysis **(B)** illustrating the protein expression levels of senescence markers in different groups, normalized to GAPDH in each lane, with the average levels in the control group set as 1. **(C)** ELISA assay showing the supernatant level of IL-17A and MCP-1 untreated or treated with H_2_O_2_ (200 μM), PCC1 (20 μM) and Secukinumab (70 nM). **(D-E)** Representative wound healing images **(D)** and migration rate analysis **(E)** showing the migration ability of LECs untreated or treated with H_2_O_2_ (200 μM), PCC1 (20 μM) and Secukinumab (70 nM). Scale bar: 50 μm. **(F-G)** Representative western blot images **(F)** and densitometric analysis **(G)** illustrating the protein expression levels of EMT related markers in different groups. Data are presented as the mean ± SEM. All analyses were conducted using a one-way ANOVA test. **p* < 0.05, ***p* < 0.01, ****p* < 0.001, *****p* < 0.0001.


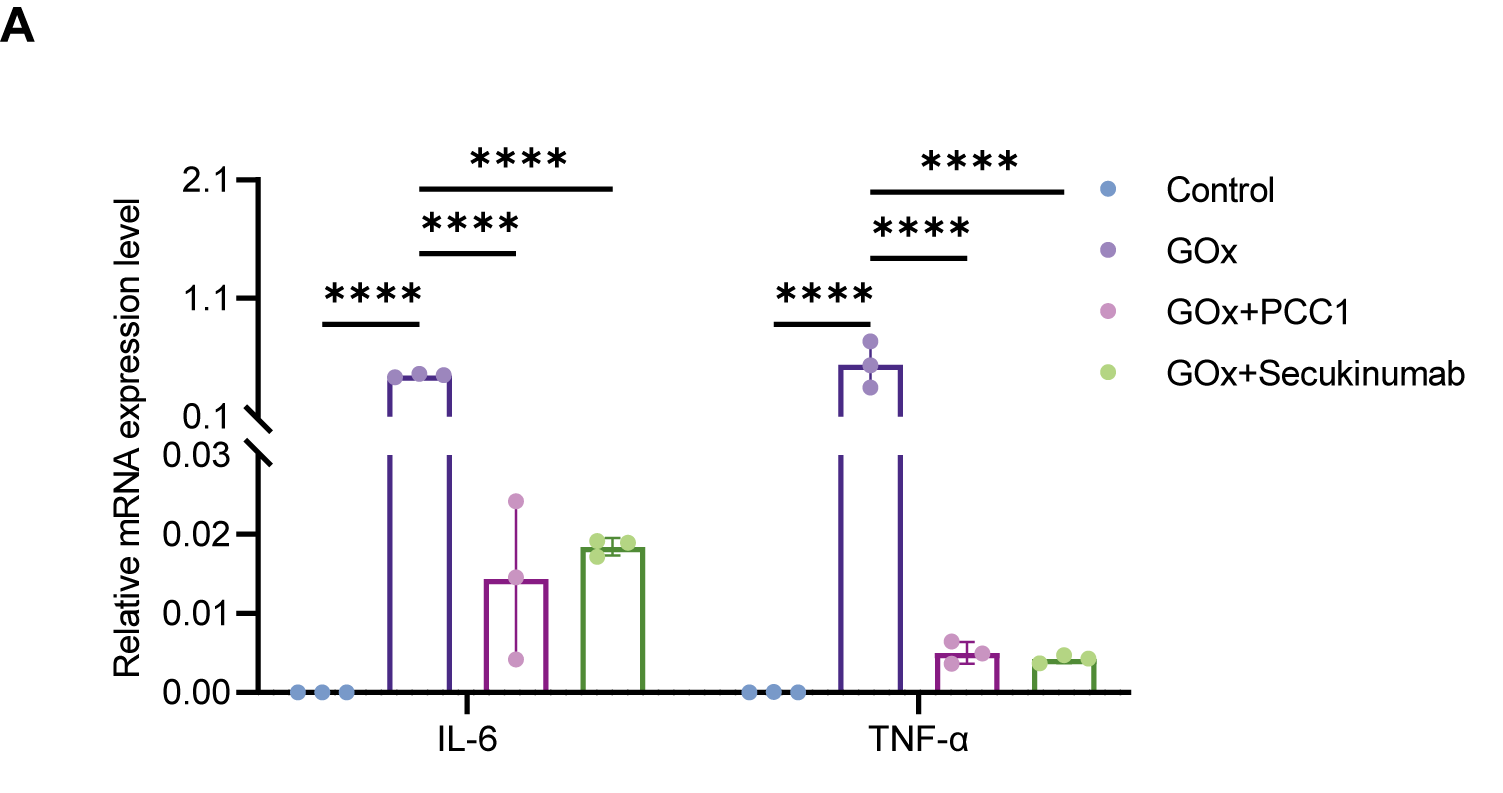


**Figure S5. PCC1 and Secukinumab suppress GOx-induced pro-inflammatory cytokines in LECs. (A)** qRT-PCR analysis of IL-6 and TNF-α mRNA levels in cultured cells from different groups. Data are presented as the mean ± SEM. All analyses were conducted using a one-way ANOVA test. *****p* < 0.0001.


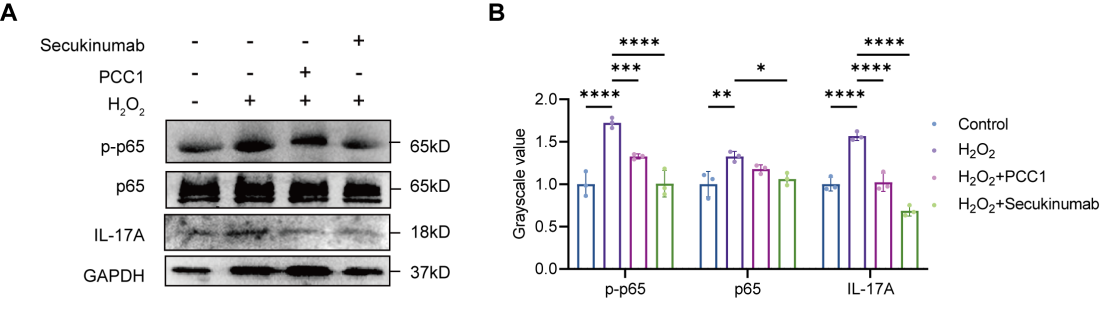


**Figure S6. PCC1 alleviated inflammaging-induced EMT in H_2_O_2_-treated cells via IL-17A/NF-κB signaling pathway. (A-B)** Representative western blot images **(A)** and densitometric analysis **(B)** illustrating the protein expression levels of IL-17A, p65 and p-p65 in different groups. Data are presented as the mean ± SEM. All analyses were conducted using a one-way ANOVA test. **p* < 0.05, ***p* < 0.01, ****p* < 0.001, *****p* < 0.0001.

**Supplementary Tables**

| **Table S1. Basic details of clear lens donors** | | | | |
| --- | --- | --- | --- | --- |
| **No.** | **Age (years)** | **Gender** | **Cause of death** | **Lens status** |
| 1 | 48 | M | Road injury | Clear |
| 2 | 50 | F | Road injury | Clear |
| 3 | 53 | M | Lung cancer | Clear |
| 4 | 55 | F | Gastric cancer | Clear |
| 5 | 52 | M | Intracerebral hemorrhage | Clear |
| 6 | 45 | F | Intracerebral hemorrhage | Clear |
| 7 | 52 | F | Intracerebral hemorrhage | Clear |
| 8 | 59 | M | Road injury | Clear |
| 9 | 47 | M | Road injury | Clear |

| **Table S2. Reagents, kits and instruments used in this study** | | | |
| --- | --- | --- | --- |
| **Category** | **Name / Description** | **Company** | **Catalog No.** |
| **Culture media & supplements** | DMEM, high glucose | Gibco (Thermo Fisher Scientific, USA) | 11965092 |
|  | Fetal bovine serum (FBS) | Gibco (Thermo Fisher Scientific, USA) | A5670701 |
|  | Penicillin–Streptomycin | Gibco (Thermo Fisher Scientific, USA) | 15140122 |
| **Reagents / Chemicals** | TRIzol reagent (RNAiso Plus) | TaKaRa Bio, Japan | 9108 |
|  | NEBNext Ultra II RNA Library Prep Kit for Illumina | New England Biolabs (NEB), USA | E7770 |
|  | Procyanidin C1 (PCC1) | MedChemExpress (MCE), China | HY-N2342 |
|  | Secukinumab | MedChemExpress (MCE), China | HY-P9927 |
|  | Recombinant human IL-17A | MedChemExpress (MCE), China | HY-P7036 |
|  | Hydrogen peroxide (H₂O₂) | Sigma-Aldrich, USA | HX0640 |
|  | Glucose oxidase | Sigma-Aldrich, USA | G2133 |
| **Kits** | Senescence β-Galactosidase Staining Kit | Beyotime, China | C0602 |
|  | Cell Counting Kit-8 (CCK-8) | TargetMol, China | C0005 |
|  | BCA Protein Assay Kit | Beyotime, China | P0012 |
|  | RIPA Lysis Buffer | Beyotime, China | P0013 |
|  | enhanced chemiluminescence (ECL) detection kit | Merck Millipore, Germany | WBKLS0500 |
|  | Taq Pro Universal SYBR Green qPCR Master Mix | Vazyme, China | Q712-02 |
|  | MILLIPLEX MAP Human Cytokine/Chemokine Magnetic Bead Panels | Merck Millipore, Germany | HCYTMAG-60K-PX318 |
|  | MILLIPLEX MAP Human Cytokine/Chemokine Magnetic Bead Panels | Merck Millipore, Germany | TGFBMAG-64K-03 |
|  | Human MCP-1 ELISA Kit | RayBiotech, USA | P13500 |
|  | Human IL-17A ELISA Kit | RayBiotech, USA | Q16552 |
|  | loop-mediated isothermal amplification (LAMP)-based detection kit | Beyotime, China | C0305S |
| **Instruments** | LightCycler 480 qPCR system | Roche, Switzerland | – |
|  | Illumina NovaSeq 6000 sequencing platform | Illumina, USA | – |
|  | Zeiss LSM880 confocal microscope | Carl Zeiss, Germany | – |
|  | Hitachi SU8010 scanning electron microscope | Hitachi High-Tech, Japan | – |
|  | Synergy H1 Hybrid Multi-Mode Reader | BioTek, USA | – |

| **Table S3. Antibodies used in the experiments** | | | | | |
| --- | --- | --- | --- | --- | --- |
| **Antibody** | **Source** | **Company** | **Catalog No.** | **Application (dilution)** | **Category** |
| p53 | mouse | CST | 2524 | WB (1:1000);  IF (1:100) | primary antibody |
| Vimentin | rabbit | CST | 5741 | WB (1:1000);  IF (1:100) | primary antibody |
| α-SMA | mouse | CST | 48938 | WB (1:300);  IF (1:100) | primary antibody |
| E-cadherin | rabbit | CST | 3195 | IF (1:100) | primary antibody |
| p27^Kip1^ | rabbit | CST | 3686 | WB (1:1000) | primary antibody |
| p16^INK4A^ | rabbit | ABclonal | A0262 | WB (1:1000);  IF (1:100) | primary antibody |
| p21^Waf1/Cip1^ | rabbit | CST | 2947 | WB (1:1000);  IF (1:100) | primary antibody |
| IL-17A | rabbit | Abcam | ab79056 | WB (0.5 µg/ml);  IF (10 µg/ml) | primary antibody |
| p65 | rabbit | CST | 8242 | WB (1:1000);  IF (1:100) | primary antibody |
| phospho-p65 | rabbit | CST | 3033 | WB (1:1000) | primary antibody |
| GAPDH | rabbit | CST | 5174 | WB (1:1000) | primary antibody |
| β-actin | rabbit | Abcam | ab8227 | WB (1:1000) | primary antibody |
| Smad2/3 | rabbit | CST | 3102 | WB (1:1000) | primary antibody |
| phospho-Smad2/3 | rabbit | CST | 8823 | WB (1:1000) | primary antibody |
| CD45 | rat | Thermo Fisher | 14-0451-82 | IF (1:100) | primary antibody |
| E-cadherin | mouse | Affinity | BF0219 | IF (1:100) | primary antibody |
| γ-H2AX | rabbit | Beyotime | C2035S | IF | DNA damage assay kit |
| Anti-rabbit IgG, HRP-linked Antibody | goat | CST | 7074 | WB (1:2000) | secondary antibody |
| Anti-mouse IgG, HRP-linked Antibody | horse | CST | 7076 | WB (1:2000) | secondary antibody |
| Alexa 488-conjugated anti-mouse antibody | goat | Bioss | bs-0296G-AF488 | IF (1:250) | secondary antibody |
| Alexa 594-conjugated anti-rabbit antibody | goat | Bioss | bs-0295G-AF594 | IF (1:250) | secondary antibody |
| Alexa 647-conjugated anti-rat antibody | donkey | Thermo Fisher | A78947 | IF (1 µg/mL) | secondary antibody |
| CST: Cell Signaling Technology, Danvers, MA, USA; ABclonal: ABclonal, Wuhan, China; Beyotime, Beyotime, Shanghai, China; Bioss: Bioss, Beijing, China; Abcam: Abcam, Cambridge, MA, USA;Thermo Fisher, Waltham, MA, USA; WB: Western blot; IF: Immunofluorescence | | | | | |

| **Table S4. Primers for qRT-PCR used in the experiments** | |
| --- | --- |
| **Gene** | **Sequence（5’-3’）** |
| GAPDH | F: GGAGCGAGATCCCTCCAAAAT |
|  | R: GGCTGTTGTCATACTTCTCATGG |
| p16^INK4A^ | F: GTGGACCTGGCTGAGGAG |
|  | R: CTTTCAATCGGGGATGTCTG |
| p21^Waf1/Cip1^ | F: GGCAGACCAGCATGACAGATTT |
|  | R: GGCGGATTAGGGCTTCCTCT |
| p27^kip1^ | F: GCCCTCCCCAGTCTCTCTTA |
|  | R: TCAAAACTCCCAAGCACCTC |
| p53 | F: GAAGACCCAGGTCCAGATGA |
|  | R: TGTTTCCTGACTCAGAGGGG |
| Vimentin | F: AGGCAAAGCAGGAGTCCACTGA |
|  | R: ATCTGGCGTTCCAGGGACTCAT |
| COL1A1 | F: GAGGGCCAAGACGAAGACATC |
|  | R: CAGATCACGTCATCGCACAAC |
| α-SMA | F: GTGTTGCCCCTGAAGAGCAT |
|  | R: GCTGGGACATTGAAAGTCTCA |
| Twist 1 | F: GTCCGCAGTCTTACGAGGAG |
|  | R: GCTTGAGGGTCTGAATCTTGCT |
| Slug | F: TGTGACAAGGAATATGTGAGCC |
|  | R: TGAGCCCTCAGATTTGACCTG |
| IL-17A | F: TCCCACGAAATCCAGGATGC |
|  | R: GGATGTTCAGGTTGACCATCAC |
| IL-17RA | F: GCTTCACCCTGTGGAACGAAT |
|  | R: TATGTGGTGCATGTGCTCAAA |
| BCL3 | F: CCGGAGGCGCTTTACTACC |
|  | R: TAGGGGTGTAGGCAGGTTCAC |
| CEBPD | F: CCCCGCCATGTACGACGAC |
|  | R: CCCGCCTTGTGATTGCTGT |
| CXCL12 | F: ATTCTCAACACTCCAAACTGTGC |
|  | R: ACTTTAGCTTCGGGTCAATGC |
| CXCL2 | F: CATCCAAAGTGTGAAGGTGAAG |
|  | R: AGCTTTCTGCCCATTCTTGA |
| SLC6A15 | F: GGAACTCTCTGTGGGTCAAAG |
|  | R: CCGCCCAGTTTAGGGCTTA |
| ITGA2 | F: CCTACAATGTTGGTCTCCCAGA |
|  | R: AGTAACCAGTTGCCTTTTGGATT |
| EGR1 | F: GGTCAGTGGCCTAGTGAGC |
|  | R: GTGCCGCTGAGTAAATGGGA |
| SOX4 | F: AGCGACAAGATCCCTTTCATTC |
|  | R: CGTTGCCGGACTTCACCTT |
| IL-6 | F: ACTCACCTCTTCAGAACGAATTG |
|  | R: CCATCTTTGGAAGGTTCAGGTTG |
| TNF-α | F: CCTCTCTCTAATCAGCCCTCTG |
|  | R: GAGGACCTGGGAGTAGATGAG |
